# Selective vulnerability of GABAergic neurons in chronic migraine

**DOI:** 10.1186/s10194-025-02223-9

**Published:** 2025-11-20

**Authors:** Kazi Helal Hossain, Timothy Chuong, Emily Abad, Justin Lin, Chenchen Xia, Meng Li, Yibu Chen, Xianghong Arakaki, Anju Vasudevan

**Affiliations:** 1 Angiogenesis and Brain Development Laboratory, Department of Neurosciences, Huntington Medical Research Institutes (HMRI), 686 S Fair Oaks Avenue, Pasadena, CA 91105 USA; 2 Cognition and Brain Integration Laboratory, Department of Neurosciences, Huntington Medical Research Institutes (HMRI), 686 S Fair Oaks Avenue, Pasadena, CA 91105 USA; 3 USC Libraries Bioinformatics, University of Southern California (USC), Health Science Campus, 2003 Zonal Ave, Los Angeles, CA 90089 USA

## Abstract

**Background:**

Migraine is the second leading cause of neurological disability and has a strong genetic component. Previous linkage studies have identified a candidate migraine susceptibility locus on chromosome Xq24-28, which harbors several GABA_A_ receptor subunit genes. Despite its inhibitory role in the central nervous system, the contribution of the GABAergic system to migraine pathophysiology remains insufficiently understood. This study elucidates the role of GABAergic neurons in chronic migraine using established rodent models. We induced basal hypersensitivity as a preclinical model of chronic migraine by administering repeated intraperitoneal injections of nitroglycerin, a well-established migraine trigger, every other day over a nine-day period. Mechanical hypersensitivity, a hallmark of migraine-associated allodynia, was assessed using von Frey filaments, before and after NTG treatment. NTG-treated animals exhibited a progressive increase in mechanical sensitivity compared to controls, consistent with the development of a chronic migraine-like state.

**Results:**

Notably, a selective reduction in GABAergic neurons was observed in male, but not female, NTG-treated mice, specifically within key brain regions associated with pain processing and psychiatric circuits, from the locus coeruleus in the brainstem through the basal forebrain (notably the amygdala) to the neocortex and hippocampus. This loss of GABAergic neurons was accompanied by elevated expression of ΔFosB, a marker of sustained neuronal activation, and increased apoptotic signaling indicated by active caspase-3 staining. Furthermore, male chronic migraine mice showed upregulation of stress-related neuropeptides, including PACAP and its receptor PAC1, as well as downstream effectors BDNF and TRK1B. Gene expression analysis revealed downregulation of GABA signaling components in the choroid plexus of the fourth ventricle, including aberrant overexpression of the chloride cotransporter NKCC1.

**Conclusion:**

These findings reveal a male-specific vulnerability of GABAergic neurons in chronic migraine and suggest a sex-dependent divergence in the underlying pathophysiological mechanisms. This highlights the critical need for sex-specific approaches to migraine research and therapeutic development.

**Supplementary Information:**

The online version contains supplementary material available at 10.1186/s10194-025-02223-9.

## Introduction

Migraine affects an estimated 36 million individuals in the United States and is the second leading cause of disability worldwide [1, 2]. Notably, sex differences are well documented, with women experiencing higher prevalence and greater migraine-related disability than men [3]. While many patients experience episodic attacks, a subset progress to chronic migraine, defined as 15 or more headache days per month, with at least 8 meeting criteria for migraine, resulting in substantial functional impairment and high healthcare utilization [4–7]. Chronic migraine is frequently accompanied by affective and cognitive disturbances, with pain catastrophizing, characterized by rumination, magnification of pain, and helplessness, emerging as a prominent psychological feature [8]. Neuroimaging studies implicate limbic and prefrontal structures, including the amygdala and frontal cortex, suggesting dysfunction in circuits that regulate emotion, stress, and memory [8, 9]. Cortical circuits undergo structural and functional reorganization in chronic migraine, contributing to altered sensory discrimination and hyperexcitability [10–12]. The hippocampus exhibits persistent plasticity and volume loss linked to pain-related cognitive and emotional dysfunction [13, 14]. The amygdala mediates pain-emotion coupling and anxiety-related behaviors [15, 16], while maladaptive locus coeruleus plasticity disrupts descending pain modulation and contributes to chronic pain states [17, 18]. Together, these regions capture complementary circuits central to migraine pathophysiology and its psychiatric manifestations, providing a focused framework to interrogate sex-dependent neural adaptations.

Gamma-aminobutyric acid (GABA), the primary inhibitory neurotransmitter in the central nervous system, plays a fundamental role in maintaining excitatory-inhibitory balance. Disruption of GABAergic signaling has been implicated across neuropsychiatric disorders [19–21] and is increasingly recognized as relevant to migraine pathophysiology [22]. Reduced inhibitory tone may lower the threshold for cortical spreading depolarization (CSD), a putative neurophysiological mechanism of migraine aura [23], and alter nociceptive processing within brainstem trigeminovascular pathways [24]. Clinical studies report altered cortical GABA levels, including decreased occipital GABA in migraine with aura [25, 26], and reduced cerebrospinal fluid GABA in chronic migraine with comorbid depression [27]. Genetic studies, including mutations in *GABRG2* and genome-wide associations with *GRIA1* and *GABRB3*, further implicate inhibitory pathways in migraine susceptibility [28, 29]. Electrophysiological evidence of cortical hyperexcitability and impaired habituation in migraineurs [12], alongside the preventive efficacy of GABA-enhancing agents such as valproate, topiramate, and benzodiazepines [30], supports a functional role for GABAergic mechanisms. Preclinical studies reinforce this concept: mice carrying the *CACNA1A* R192Q familial hemiplegic migraine mutation exhibit reduced GABA release and increased CSD susceptibility [31, 32], while targeted activation of parvalbumin interneurons via chemogenetic or optogenetic approaches can suppress CSD [33]. Together, these findings underscore the critical role of GABAergic neurons in regulating cortical excitability and implicate GABAergic dysfunction as a potential shared substrate contributing to persistent pain, cognitive impairment, and affective burden characteristic of migraine.

Despite these advances, important gaps remain. Reports of GABA alterations vary across brain regions and migraine phases, with some studies showing increases, decreases, or no change [22, 34–37]. Most research focuses on episodic migraine, with limited exploration of chronic migraine or sex-specific mechanisms. Furthermore, although neurosteroids can modulate GABA_A_ receptors [38], their contribution to migraine-related circuitry remains poorly understood. The specific GABAergic circuits driving migraine onset, propagation, and associated affective comorbidities are largely undefined.

**Fig. 1:**
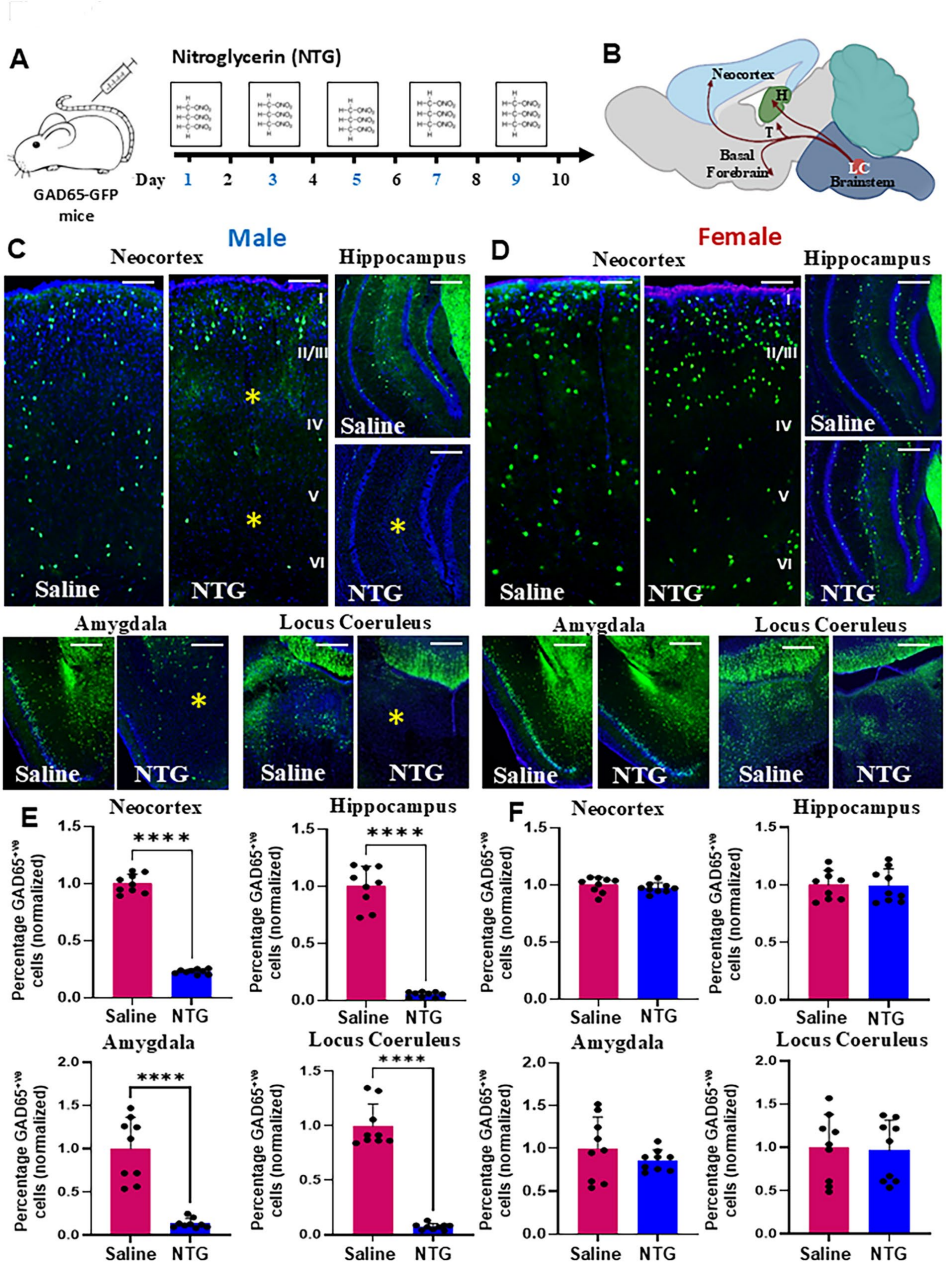
Sex-specific reduction of GABAergic neurons in NTG-treated GAD65-GFP mice. (**A**) Experimental paradigm: GAD65-GFP mice received intraperitoneal injections of either nitroglycerin (NTG, 10 mg/kg) or saline every other day for 9 days. (**B**) Schematic depiction of the locus coeruleus (LC) and its anatomical connections with key pain-processing regions and psychiatric circuits including the amygdala, neocortex, and hippocampus. (**C**) Representative low-magnification fluorescent images showing a marked reduction in GFP^+^ GABAergic neurons (indicated by asterisks) in the neocortex, hippocampus, amygdala, and LC of NTG-treated male mice compared to saline-treated controls. (**D**) In contrast, no overt changes in GABAergic neuron distribution were observed in the same brain regions of NTG-treated female mice relative to saline-treated controls. (**E**) Quantification reveals a significant reduction in the number of GABAergic neurons across all 4 analyzed brain regions in NTG-treated males. Data represents mean ± SD (*n* = 9 sections from 3 mice, ****P < 0.0001, Student’s t-test). (**F**) Quantitative analysis in females shows no statistically significant differences between NTG- and saline-treated groups. Scale bars: 100 μm (**C**; applies to **D**)

To address these gaps, we employed complementary mouse and rat models of chronic migraine (Fig. 1A) with the use of nitroglycerin (NTG). NTG is a known migraine trigger and is used as a human experimental model of migraine [39]. Acute administration of NTG has been used to model preclinical acute migraine [40, 41] while recurrent NTG-induced basal hypersensitivity has been used to model preclinical chronic migraine [42, 43]. Using GAD65-GFP transgenic mice [44] we mapped GABAergic neuronal alterations across chronic migraine-relevant brain regions, while parallel rat studies enabled molecular profiling of choroid plexus signaling. We focused on the neocortex, hippocampus, amygdala, and locus coeruleus because these regions constitute key sensory, affective, and neuromodulatory hubs implicated in pain chronification and pain-psychiatric comorbidities (Fig. 1B). Our results reveal previously unrecognized, sex-specific alterations in GABAergic circuits and stress-related pathways in these brain regions, providing novel insights into the neurobiological mechanisms of chronic migraine and offering potential avenues for targeted therapeutic interventions.

## Materials and methods

### Animals

Chronic migraine was modeled in two species: transgenic GAD65-GFP mice - generously provided by Dr. Gabor Szabo - which express green fluorescent protein (GFP) specifically in GABAergic neurons [44, 45], and Sprague-Dawley rats (~ 2-3-month old, *n* = 12 total: 6 males and 6 females) obtained from Envigo. GAD65-GFP mice were housed in the HMRI animal facility under standard conditions, including a 12-hour light/dark cycle with unrestricted access to food and water. Both male and female adult animals were included in the study. All animal procedures were conducted in accordance with the NIH Guide for the Care and Use of Laboratory Animals and were approved by the HMRI Institutional Animal Care and Use Committee. All procedures were conducted in accordance with ARRIVE 2.0 guidelines.

### Nitroglycerin administration

Nitroglycerin (American Regent) was used in this study. For experimental use, NTG solution at a dose of 10 mg/kg was administered via intraperitoneal (i.p.) injection to adult mice or rat (~ 2-3-month old) every other day over a nine-day period, for a total of five injections. The stock solution of nitroglycerin contained 30% alcohol, 30% propylene glycol, and water. NTG was freshly diluted in 0.9% saline to a dose of 10 mg/kg. Control animals received equivalent volumes of 0.9% saline [42] via i.p. injection following the same schedule. This dosage of NTG and usage of saline as a control has been well-established in chronic migraine studies [42, 46].

### GAD65-GFP mice and experimental design

Adult GAD65-GFP mice (*n* = 12 total; 6 males and 6 females) were used. Mice were randomly assigned to receive either nitroglycerin (NTG, 10 mg/kg, i.p; *n* = 6) to induce basal hypersensitivity, or saline as a control (*n* = 6). Each treatment group included 3 males and 3 females, maintaining a balanced sex ratio. To minimize potential litter effects, no more than 2 mice from any litter were used in a given experiment, and treatment groups were assembled from 5 to 7 independent litters, ensuring that both NTG and saline groups were balanced across litters. Details of sample size calculations and power analysis are provided in Supplementary Table 1.

### Mechanical sensitivity (von Frey) test

In this method, a series of monofilaments were applied to the plantar surface of the hind paw while the animal was resting on a mesh screen, and basal thresholds and post-treatment aversion thresholds were evaluated using established methodology [43]. Mechanical hypersensitivity, a hallmark of migraine-associated allodynia, was assessed using von Frey filaments both before and after nitroglycerin (NTG) administration. Behavioral testing included both male and female mice. Prior to evaluation, animals were acclimated to the testing environment for 1 h. Mechanical sensitivity was measured using standard protocols [42, 43]. Mice were individually placed in a four-chamber apparatus constructed of transparent Plexiglas positioned over an elevated wire mesh platform. Von Frey filaments were applied from below the mesh to the plantar surface of the hind paw. A positive response was defined as a reflexive paw withdrawal - such as flicking or lifting - indicating the detection of a mechanical threshold. Filaments of increasing stiffness were applied sequentially until a withdrawal response was elicited. Baseline mechanical responses were recorded after habituation and prior to NTG or saline injection. Post-treatment sensitivity was assessed 2 h following injection. Sample sizes for each group are indicated in the corresponding figure legends.

### Tissue processing

After the completion of the von Frey test, on day 9, mice were anesthetized with ketamine (100 mg/kg i.p.) and xylazine (10 mg/kg i.p.) and perfused via the heart with 4% paraformaldehyde (PFA; BD Biosciences PharMingen). The brains were dissected from the skull and placed in the same fixative solution for 24 h at 4 °C. Next, brains were cryoprotected in 15% and 30% sucrose solutions, frozen on dry ice, and stored at -80 °C.

### Immunohistochemistry and microscopic analysis

Frozen immunohistochemistry (IHC) was performed on mouse brain tissue. Cryoprotected, frozen brains were embedded in optimal cutting medium and sectioned at 30 μm using a cryostat. The following primary antibodies were used: anti-ΔFosB (1:50, Cell Signaling Technology), anti-PACAP (1:200, Thermo Fisher), anti-PAC1 (1:200, Thermo Fisher), anti-BDNF (1:200, Abcam), anti-TRK1B (1:200, Sigma), anti-NKCC1 (1:200, Thermo Fisher), anti-KCC2 (1:100, Thermo Fisher), anti-active caspase-3 (1:200, Sigma), and anti-GAD65/67 (1:100, Sigma). Secondary detection was performed using Alexa Fluor-conjugated antibodies (Invitrogen). Nuclei were counterstained with DAPI (Vector Laboratories). Fluorescent images were acquired at both low and high magnification using either the FSX100 microscope (Olympus) or the BZ-X800 All-In-One Fluorescence Microscope (Keyence).

### GAD65-GFP cell quantification

GAD65-GFP⁺ cells were quantified in the neocortex, hippocampus, amygdala (at bregma − 1.5), and locus coeruleus (LC; at bregma − 5.41) in both the left and right hemispheres, using stereotaxic coordinates from Paxinos and Franklin’s - The Mouse Brain in Stereotaxic Coordinates. For each region, GFP⁺ cells were counted within defined anatomical boundaries - layer I to VI in the neocortex, and established neuroanatomical landmarks in the hippocampus, amygdala, and LC. Cell counts were performed using ImageJ, and data were plotted for comparative analysis across experimental groups.

### Gene expression profile analysis

Gene expression analysis in rats was performed on a chronic migraine model derived from naïve animals. Choroid plexus tissue from fourth ventricle of adult male and female rats was collected on DNA-free petri dishes on ice, two hours after the final NTG or saline injection, and immediately stored in RNAlater within DNA-free Eppendorf tubes and stored at − 80 °C. RNA was extracted from choroid plexus using PicoPure RNA Isolation kit (Arcturus) following the supplier’s protocol. RNA quality was determined and RNA-seq was performed by Quick Biology Inc, 800 Royal Oaks Drive, Monrovia, CA. Samples were subjected to 150 cycle pair-end sequencing using Illumina Sequencers. Reads were demultiplexed using Illumina bcl2fastq2 v2.20 with default settings. Read quality was assessed using FastQC. Raw sequencing files were transferred to the USC Libraries Bioinformatics Services for further analysis using Partek Flow software. For RNA-seq analyses, RNA-seq data was analyzed primarily using the RNA-seq workflow in Partek Flow software (v11, Illumina, San Diego, CA, USA), (Partek Inc., St. Louis, MO). Briefly, the raw sequencing reads were first trimmed based on the quality score (Phred QC >= 30, min read length = 25 nt) before being mapped to the rat genome build rn7 with Star aligner (2.7.8a [47]). Ensembl v111 annotation [48] was used to quantify the aligned reads using Partek E/M methods. The quantified reads were normalized and analyzed for differential gene expression using DESeq2 [49] as implemented in Partek Flow (genes/transcripts with 10 reads or fewer in any sample were excluded). Principal Component Analysis was used to examine and visualize the transcriptome differences among different groups, as well as among the samples within the same group. The differentially expressed gene (DEG) lists were generated for each comparison using the cutoff of FDR < 0.05 and fold change cutoff of +/- 2. Subsequent functional analysis of the DEG lists were conducted using Ingenuity Pathway System (Qiagen, Redwood City, CA, USA).

### Statistical analysis

Analyses were performed separately by sex. For between-group comparisons we used two-tailed Student’s *t*-test, with results confirmed using the non-parametric Mann-Whitney U test (GraphPad Prism); both methods produced consistent outcomes and are reported in the Results. Statistical significance was defined at *P* < 0.05. Data are presented as mean ± SD, and individual data points are shown in the figures; figure legends specify the tests used (Student’s *t*-tests).

Tissue data spanning multiple brain regions were evaluated using linear mixed-effects models to account for within-subject correlation across regions. In addition, a two-way ANOVA with factors Treatment and Region was performed for each outcome, and pairwise post-hoc contrasts were adjusted using Tukey’s test. To control the false discovery rate across multiple comparisons, Benjamini-Hochberg correction was applied across all readout × region pairwise tests (Supplementary Tables 2–8).

### Randomization and blinding

Animals were randomized to treatment groups with balanced sex ratios. Tissue processing, imaging, and image analysis were conducted under blinded conditions. Slides were coded by independent personnel, and investigators analyzing the data were unaware of group assignments.

## Results

### Unexpected sex-specific vulnerability in GABAergic neurons in chronic migraine

To model chronic migraine-associated pain, NTG was administered systemically every second day for 9 days (5 i.p. injections total, each of ~ 100 µl) to adult GAD65-GFP mice (Fig. 1A). Half of the animals received saline injections (controls), and the other half received NTG. On test days, basal responses were assessed before NTG administration, and post-treatment responses were determined two hours after NTG injection. Briefly, mechanical sensitivity was determined using manual von Frey hair stimulation (Supplementary Fig. 1A, B). Each injection of NTG injection results in a significantly reduced threshold within 2 h of administration (post-treatment responses). In addition, repeated injections of NTG produce a progressive decrease in basal mechanical responses (basal responses). By day 9, the basal response for NTG treated GAD65-GFP mice were almost as low as after NTG administration (Supplementary Fig. 1C, D). The spontaneous grimace, a sign of nociceptive response was observed in NTG-treated GAD65-GFP mice, but not saline-treated controls (Supplementary Fig. 2). The interpretation of these results is that the acute NTG-evoked hyperalgesia (post-treatment responses) models an acute migraine attack, whereas the basal hypersensitivity models the progression of migraine from an episodic to chronic state.

Interestingly, a significant reduction in GAD65-GFP^+^ cells was observed in the neocortex, hippocampus, amygdala, and locus coeruleus in NTG-treated males versus saline-treated males (Fig. 1C) that was quantified (Fig. 1E; *P < 0.0001, in both Student’s *t*-test and Mann-Whitney U test, in all 4 regions). The GAD65-GFP^+^ cellular data across all 4 brain regions in males were also analyzed using linear mixed-effects models and significant reductions in the NTG versus saline groups were observed (Supplementary Table 2). On the other hand, GAD65-GFP^+^ cells in NTG-treated females were comparable to female controls (Fig. 1D, F). GAD65/67 immunohistochemistry in NTG-treated males, females, and controls showed similar reductions selectively in the male-NTG-treated group (Supplementary Fig. 3).

### Coordinate region-specific increase in neuronal activation and apoptosis in male chronic migraine

**Fig. 2:**
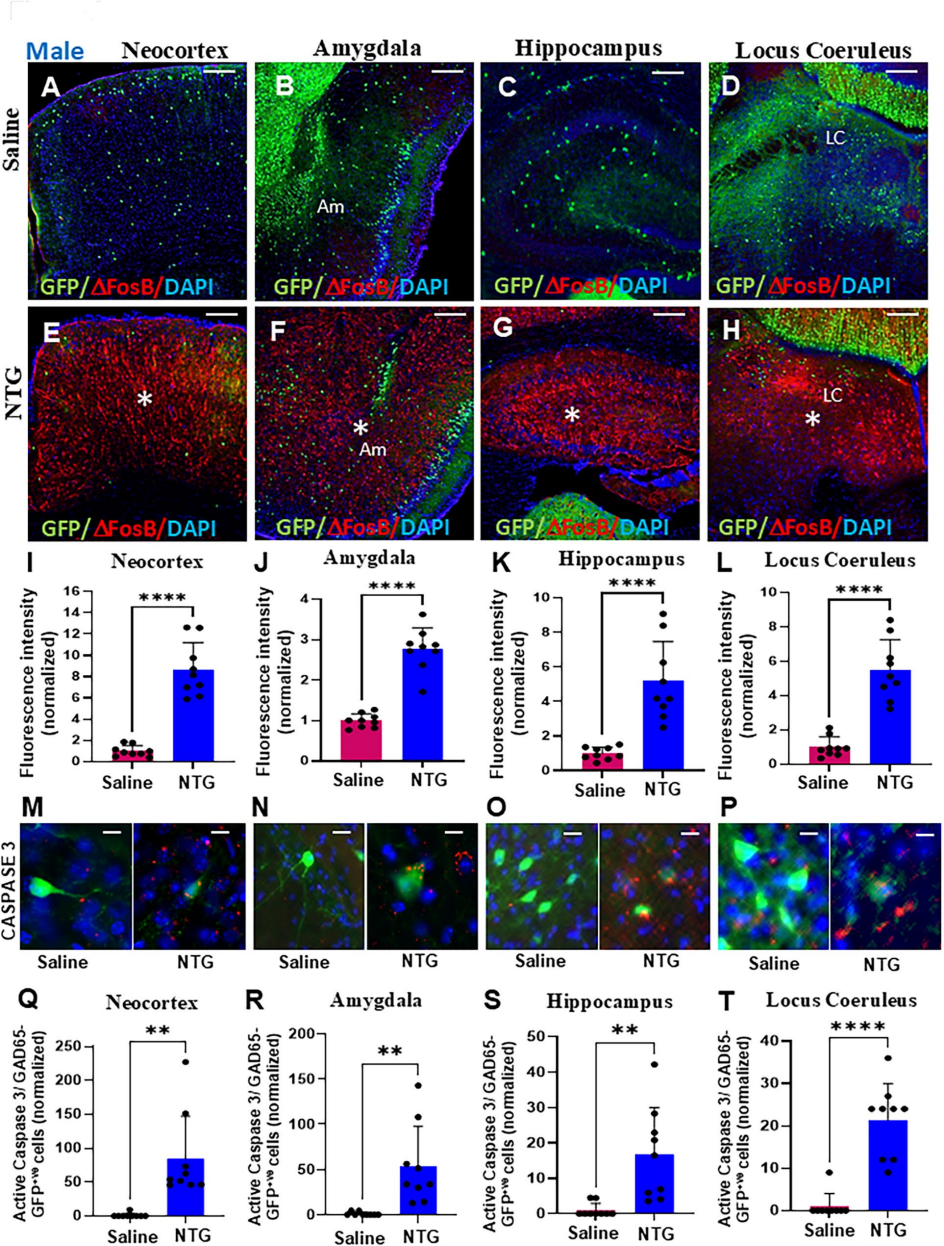
Region-specific increases in neuronal activation and apoptosis in male mice in a chronic NTG migraine model. (**A**-**H**) Representative low-magnification images illustrating elevated ΔFosB expression (marked by asterisks) in the neocortex, amygdala, hippocampus, and locus coeruleus (LC) of NTG-treated male GAD65-GFP mice compared to saline-treated controls. (**I**-**L**) Quantitative analysis confirms a significant increase in ΔFosB-positive cells in the neocortex (**I**), amygdala (**J**), hippocampus (**K**), and LC (**L**) of NTG-treated males (*n* = 9 sections from 3 mice, ****P < 0.0001, Student’s t-test). (**M-P**) High-magnification images showing co-localization of active caspase-3 (an apoptotic marker) with GAD65-GFP-positive neurons in the neocortex (**M**), amygdala (**N**), hippocampus (**O**), and LC (P). (**Q**-**T**) Quantification indicates a significant increase in apoptotic GABAergic neurons in NTG-treated males (*n* = 9 sections from 3 mice; **P = 0.0012, Neocortex, Q; **P = 0.0025, Amygdala, R; **P = 0.0025, Hippocampus, S, ****P < 0.0001, Locus Coeruleus, T; Student’s t-test). Scale bars: 100 μm (**A**; applies to **B**-**H**), 50 μm (**M**; applies to **N**-**P**). Am: Amygdala, LC: Locus coeruleus

We next questioned whether the region-specific reduction in GABAergic neurons observed in male NTG-treated mice was accompanied by changes in neuronal activation, and/or cell death. Delta FosB (ΔFosB) expression has been reported to be increased in chronic pain models, but not in acute pain models, supporting that ΔFosB may be an important transcriptional factor that modulates neuronal adaptation specifically to pain resulting from chronic stress, suggesting chronic activation of neurons. Therefore, we explored ΔFosB expression in our GAD65-GFP chronic migraine model by immunohistochemistry. Expression of ΔFosB was significantly increased in the neocortex, amygdala, hippocampus, and locus coeruleus of NTG-treated males (Fig. 2E-H), versus saline-treated males (Fig. 2A-D) that was quantified (Fig. 2I-L, *P < 0.0001, in both Student’s *t*-test and Mann-Whitney U test, in all 4 regions). The ΔFosB data across all 4 brain regions in males were also analyzed using linear mixed-effects models, and significant increases in the NTG versus saline groups were observed in the neocortex, hippocampus, and locus coeruleus. However, in the amygdala, significance was observed only with the Benjamini-Hochberg FDR correction (Supplementary Table 3). ΔFosB expression was comparable between saline and NTG-treated female mice (Supplementary Fig. 4).

We next evaluated for hallmarks of apoptosis with active caspase 3 that triggers cell death pathways, in a defined cascade of events ultimately leading to regulated death. A significant increase in active caspase 3 labeling was observed in GAD65-GFP^+^ cells in the NTG-treated male group versus saline treated male controls (Fig. 2M-P) that was quantified in the neocortex (Fig. 2Q, *P = 0.0012, in Student’s *t*-test and *P < 0.0001 in Mann-Whitney U test), amygdala (Fig. 2R, *P = 0.0025, in Student’s *t*-test and *P < 0.0001 in Mann-Whitney U test), hippocampus (Fig. 2S, *P = 0.0025, in Student’s *t*-test and *P = 0.0004 in Mann-Whitney U test), and locus coeruleus (Fig. 2T, *P < 0.0001 in both Student’s *t*-test and Mann-Whitney U test). The active caspase 3 data across all 4 brain regions in males were also analyzed using linear mixed-effects models, and significant increases in the NTG versus saline groups were observed only in the neocortex, and amygdala (Supplementary Table 4). Active caspase 3 expression was significantly increased in the neocortex, amygdala, hippocampus, and locus coeruleus in the NTG-treated male group versus the saline group (Supplementary Fig. 5).

### Increase in stress peptides and downstream targets in male chronic migraine

**Fig. 3:**
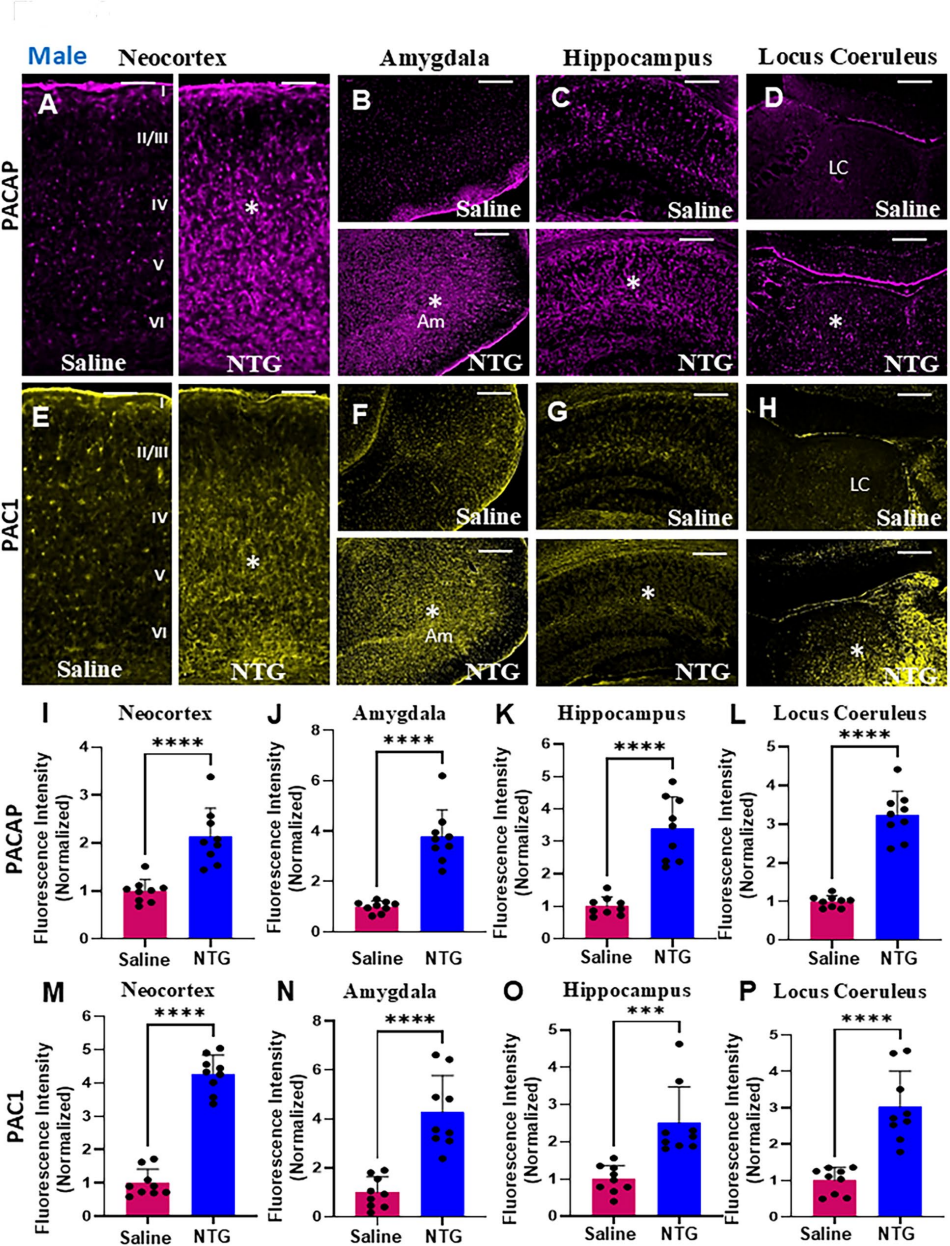
PACAP and PAC1 receptor are upregulated in male mice in a chronic NTG migraine model. (**A**-**D**) Representative low-magnification images showing increased expression of the stress peptide PACAP (indicated by asterisks) in the neocortex (**A**), amygdala (**B**), hippocampus (**C**), and locus coeruleus (LC) (**D**) of NTG-treated male mice compared to saline-treated controls. (**E**-**H**) Representative images demonstrate elevated expression of PAC1⁺ cells (asterisks) in the neocortex (**E**), amygdala (**F**), hippocampus (**G**), and LC (**H**) of NTG-treated males. (**I**-**L**) Quantification confirms a significant increase in PACAP expression in the neocortex (**I**), amygdala (**J**), hippocampus (**K**), and LC (**L**) following NTG treatment (*n* = 9 sections from 3 mice, ****P < 0.0001, Student’s t-test). (**M–P**) Quantification of PAC1⁺ cells also reveal a significant elevation in the neocortex (**M**), amygdala (**N**), hippocampus (**O**), and LC (**P**) of NTG-treated males compared to controls (*n* = 9 sections from 3 mice, ****P < 0.0001, Student’s t-test, neocortex, M; amygdala, N; and locus coeruleus, P; ***P = 0.0005, Student’s t-test, hippocampus, **O**). Scale bars: 100 μm (**A**; applies to **A**-**H**). Am: Amygdala, LC: Locus coeruleus

We next questioned whether stress peptides were altered in specific brain regions as a consequence of increased neuronal activation or apoptosis, by immunohistochemistry. The stress response in the adult brain is orchestrated through intricate signaling networks involving neuropeptides, neurotransmitters, and their receptors. Among these, Pituitary Adenylate Cyclase-Activating Polypeptide (PACAP), and its high-affinity receptor PAC1 have emerged as pivotal regulators of stress adaptation, emotional behavior, and neuroplasticity. A significant increase in PACAP (Fig. 3A-D) and PAC1 (Fig. 3E-H) expression were observed in male NTG-treated mice in the neocortex, amygdala, hippocampus, and locus coeruleus in comparison to controls. The increase in PACAP expression was quantified (Fig. 3I-L; *P < 0.0001, in both Student’s *t*-test and Mann-Whitney U test, in all 4 regions). The increase in PAC1 expression was also quantified (Fig. 3M, N, P; *P < 0.0001, in both Student’s *t*-test and Mann-Whitney U test, in neocortex, amygdala, and locus coeruleus; Fig. 3O; *P = 0.0005, in Student’s *t*-test and *P < 0.0001 in Mann-Whitney U test). PACAP1 and PAC1 expression across all 4 brain regions in males were also analyzed using linear mixed-effects models and a significant increase in the NTG versus saline groups were observed (Supplementary Tables 5 and 6). PACAP expression in brain regions of female NTG treated mice was comparable to controls (Supplementary Fig. 6).

**Fig. 4:**
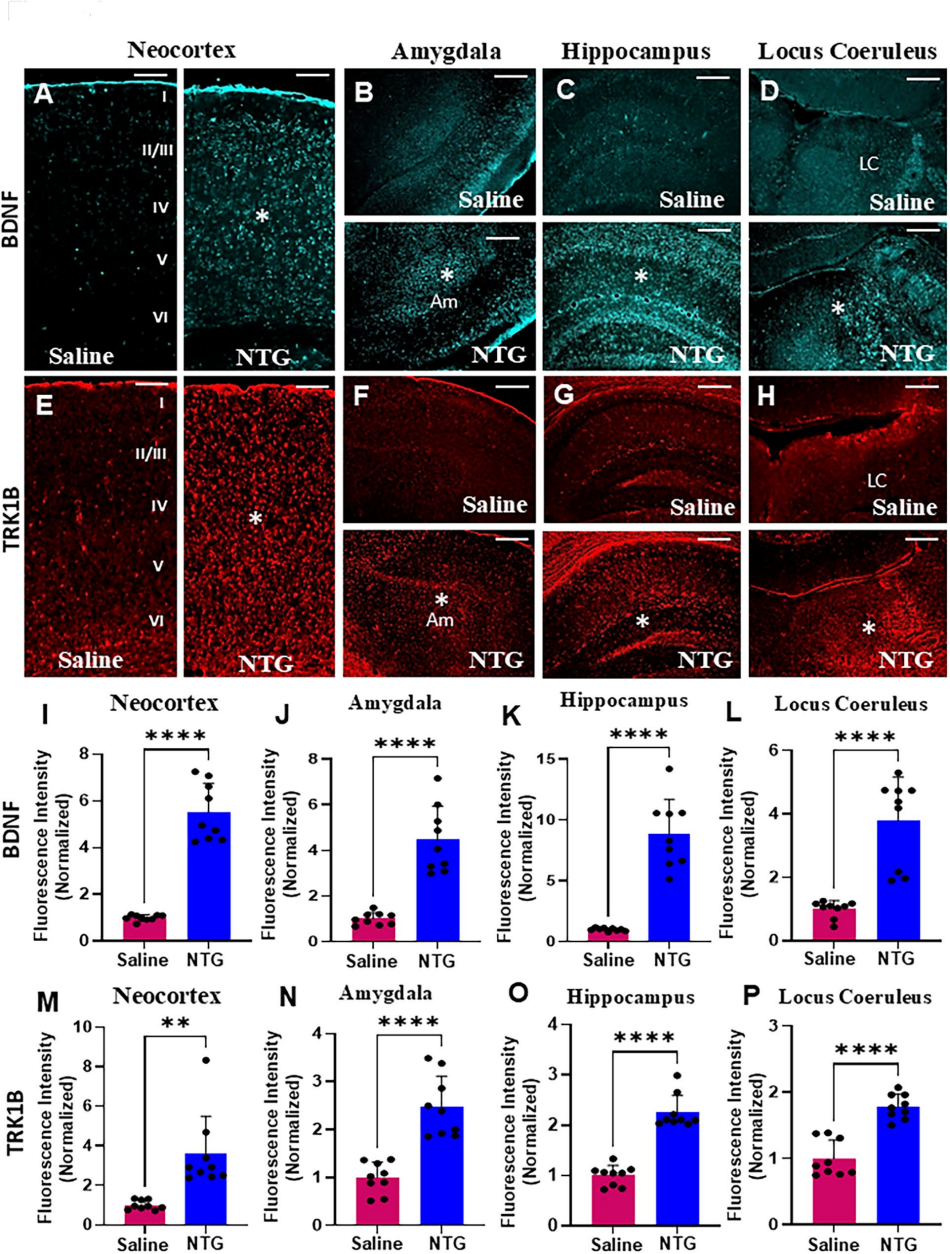
Stress-related effectors BDNF and TRK1B are upregulated in male mice in a chronic NTG migraine model. (**A**-**D**) Representative low-magnification images showing increased expression of the stress-responsive neurotrophin BDNF (indicated by asterisks) in the neocortex (**A**), amygdala (**B**), hippocampus (**C**), and locus coeruleus (LC) (**D**) of NTG-treated male mice compared to saline-treated controls. (**E**-**H**) Representative images depicting elevated expression of TRK1B (the high-affinity BDNF receptor) in the neocortex (**E**), amygdala (**F**), hippocampus (**G**), and LC (**H**) following NTG treatment. (**I**-**L**) Quantitative analysis confirms a significant increase in BDNF expression in the neocortex (**I**), amygdala (**J**), hippocampus (**K**), and LC (**L**) of NTG-treated males (*n* = 9 sections from 3 mice, ****P < 0.0001, Student’s t-test). (**M–P**) TRK1B quantification also reveals a significant upregulation in the neocortex (**M**, **P = 0.0010, Student’s t-test), amygdala (**N**, ****P < 0.0001, Student’s t-test), hippocampus (**O**, ****P < 0.0001, Student’s t-test), and LC (**P**, ****P < 0.0001, Student’s t-test) compared to saline-treated controls (*n* = 9 sections from 3 mice). Scale bars: 100 μm (**A**; applies to **A**-**H**). Am: Amygdala, LC: Locus coeruleus

In addition to its role in stress response, PACAP contributes to neuronal plasticity, potentially via crosstalk with neurotrophic factors like brain-derived neurotrophic factor (BDNF). PACAP signaling intersects significantly with BDNF and its receptor tyrosine receptor kinase B TRK1B, forming a key neurotrophic–neuropeptide axis involved in adaptive and maladaptive stress responses. Therefore, we evaluated whether downstream targets BDNF, and TRK1B were concurrently affected in brain regions affected by chronic migraine. Again, we found a significant increase in BDNF (Fig. 4A-D) and TRK1B (Fig. 4E-H) expression in the neocortex, amygdala, hippocampus, and locus coeruleus only in male NTG-treated mice when compared to controls. The increase in BDNF expression was quantified (Fig. 4I-L; *P < 0.0001, in both Student’s *t*-test and Mann-Whitney U test, in all 4 regions). The increase in TRK1B expression was also quantified (Fig. 4M, *P = 0.0010, in Student’s *t*-test and *P < 0.0001 in Mann-Whitney U test, in the neocortex and Fig. 4N-P; *P < 0.0001, in both Student’s *t*-test and Mann-Whitney U test, in amygdala, hippocampus, and locus coeruleus). BDNF and TRK1B expression across all 4 brain regions in males were analyzed using linear mixed-effects models and a significant increase in the NTG versus saline groups were observed (Supplementary Tables 7 and 8). For the TRK1B data in the locus coeruleus, significance was observed only with the Benjamini-Hochberg FDR correction (Supplementary Table 8). BDNF expression in brain regions of female NTG-treated mice were comparable to controls (Supplementary Fig. 7).

### Sex-specific differences in GABA pathway gene expression in the choroid plexus in male chronic migraine

To investigate whether GABAergic signaling pathways are differentially affected in the choroid plexus during chronic migraine, we adopted a rat NTG model, which provides a larger and more anatomically accessible fourth ventricle choroid plexus compared to mice (4–6 times greater volume). Following chronic NTG or saline treatment, we microdissected the fourth ventricle choroid plexus from male and female rats and performed bulk RNA sequencing (RNA-seq). Differential gene expression analysis and downstream pathway enrichment using Ingenuity Pathway Analysis (IPA) were conducted to identify sex-specific transcriptional changes.

**Fig. 5:**
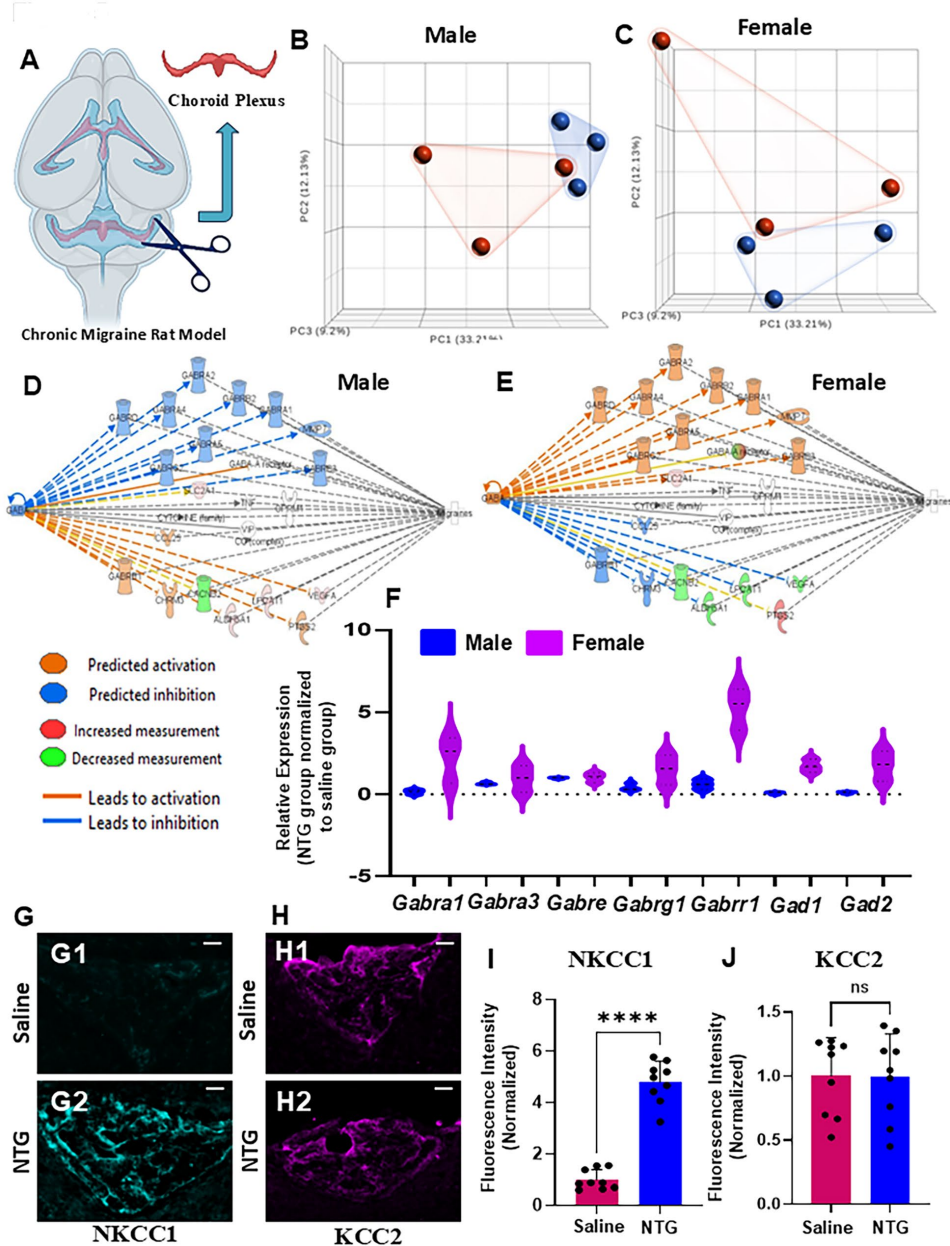
Sex-specific regulation of GABA signaling pathways in the fourth ventricle choroid plexus in a chronic NTG migraine model. (**A**) Schematic illustrates the anatomical location of the choroid plexus in the fourth ventricle, the region used for molecular and histological analysis. (**B**, **C**) Principal component analysis (PCA) reveals distinct clustering of overall transcriptomic profiles between NTG-treated (blue) and saline-treated (red) groups, with tight grouping of three independent biological replicates in both males (**B**) and females (**C**), indicating robust and consistent gene expression changes. (**D**) Functional pathway analysis of differentially expressed genes (DEGs) using Ingenuity Pathway Analysis (IPA) shows significant inhibition of GABAergic signaling components in the choroid plexus of NTG-treated males relative to controls. (**E**) In contrast, GABAergic signaling components were activated in the choroid plexus of NTG-treated females compared to saline-treated controls. (**F**) Violin plots illustrate this sex-specific regulation, highlighting the downregulation of key GABA signaling genes - *Gad1*, *Gad2*, and GABA receptor subunits - in males, and their upregulation in females as identified by IPA. (**G**, **H**) To assess downstream GABA signaling, immunohistochemical analysis was performed using anti-NKCC1 (aqua) and anti-KCC2 (purple) antibodies. Representative image (**G**) shows a marked increase in NKCC1 expression in the fourth ventricle choroid plexus of NTG-treated males (G2) relative to saline-treated controls (G1), as quantified in (**I**). (**H**) In contrast, KCC2 expression was unchanged between NTG (H2) and saline (H1) groups, with quantification shown in (**J**). Data represents mean ± SD (*n* = 9 sections from 3 mice; ****P < 0.0001, Student’s t-test). Scale bars: 50 μm (**G**; applies to **H**)

Principal component analysis (PCA) revealed clear separation between NTG-treated and control groups in both sexes, indicating robust transcriptional reprogramming (Fig. 5B-C). Strikingly, IPA indicated inhibition of GABA signaling pathways in NTG-treated males (Fig. 5D; Supplementary Fig. 8A), whereas the same pathways were activated in NTG-treated females (Fig. 5E; Supplementary Fig. 8B). Key GABA-related genes, including *Gabra1*,* Gabra3*,* Gabre*,* Gabrg1*,* Gad1*, and *Gad2*, displayed opposing regulation between sexes when normalized to respective saline controls, as illustrated in violin plots (Fig. 5F). These findings suggest that the choroid plexus engages fundamentally divergent GABAergic adaptations in males versus females during chronic migraine.

**Fig. 6:**
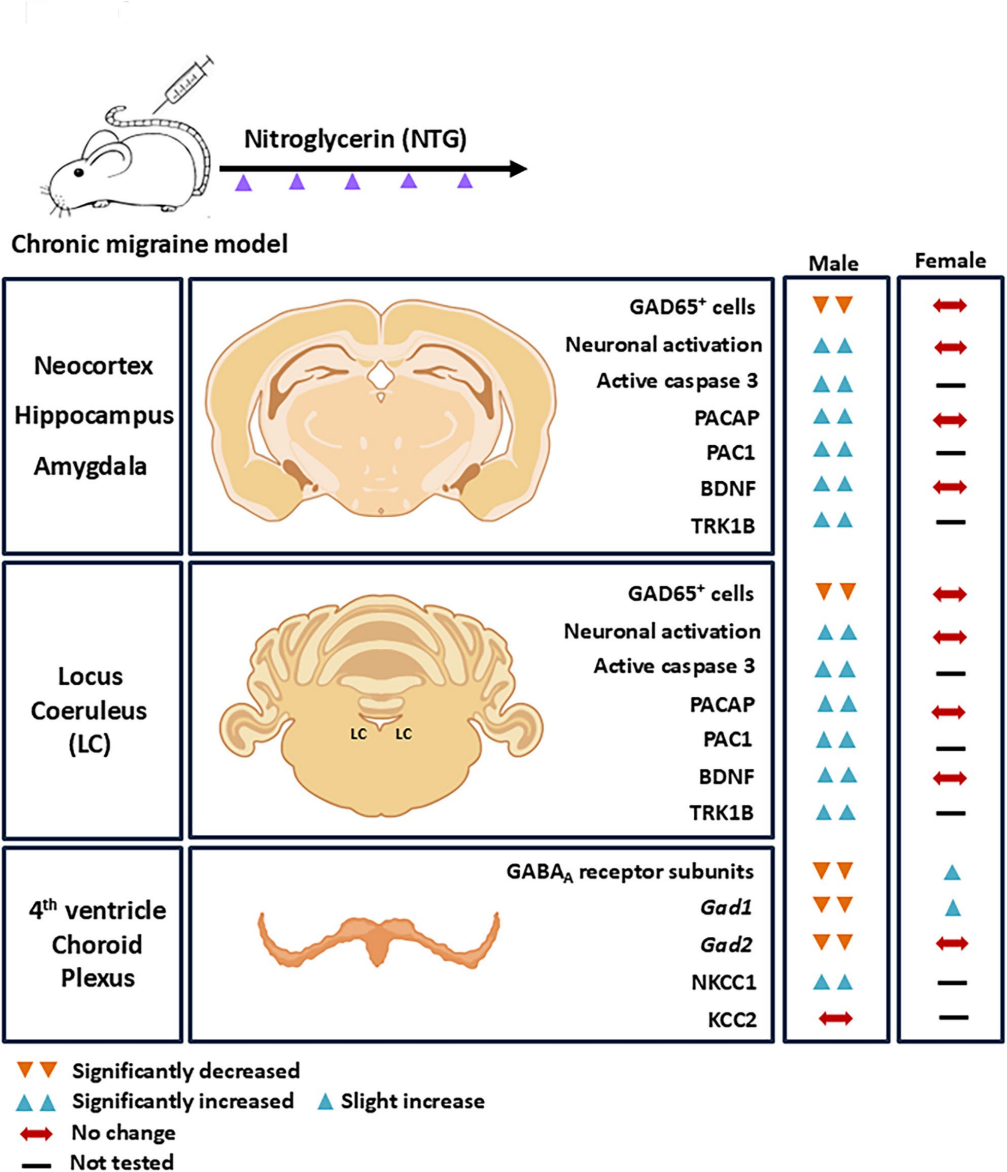
Summary of sex differences in neuropathology in a chronic NTG migraine mouse model. Brain regions implicated in pain processing and psychiatric circuits - including those receiving projections from the brainstem locus coeruleus through the basal forebrain (notably the amygdala) to the neocortex and hippocampus - along with the choroid plexus of the fourth ventricle, were systematically examined for cellular and molecular markers listed. Double inverted orange triangles - significantly decreased, double upright blue triangles - significantly increased, single upright blue triangle - slight increase, bidirectional dark red arrow - no change, black straight line - not tested

Disruption of chloride homeostasis through altered NKCC1 or KCC2 activity may impair GABAergic inhibition, promoting neuronal hyperexcitability, facilitating cortical spreading depolarization (CSD), and driving central sensitization, key processes in migraine pathophysiology. Therefore, we assessed NKCC1 and KCC2 expressions in the choroid plexus of the fourth ventricle of NTG and saline-treated mice. NKCC1 expression was significantly increased in NTG-treated males versus controls (Fig. 5G, I, *P < 0.0001, in both Student’s *t*-test and Mann-Whitney U test), indicating impaired chloride homeostasis. KCC2 expression in NTG treated males was comparable to controls (Fig. 5H, J). Similar observations in NKCC1/KCC2 expression were seen in the choroid plexus of the lateral ventricle in NTG-treated males versus controls (Supplementary Fig. 9). Combined, these results implicate sex-specific differences in GABA pathway gene expression in the choroid plexus in male chronic migraine.

## Discussion

Our findings uncover a series of novel and mechanistically important insights into chronic migraine, particularly highlighting sex-specific vulnerabilities within inhibitory GABAergic systems. First, we reveal a striking heterogeneity in GABAergic neuronal responses between males and females. In male chronic migraine models, GABAergic neurons exhibited selective degeneration across key limbic and pain-processing regions, including the locus coeruleus, amygdala, neocortex, and hippocampus. This aligns with rodent studies of neuropathic and ischemic pain demonstrating that GABAergic interneuron loss in the spinal dorsal horn drives central sensitization and persistent pain. In the chronic post-ischemic pain (CPIP) model, oxidative stress-induced apoptosis of GABAergic neurons in laminae I-II is reversed by antioxidant treatment, mitigating pain behaviors [50]. Similarly, spinal cord injury leads to caspase-3-dependent loss of GABAergic interneurons and reduced expression of inhibitory markers such as GAD65/67 and GAT1, resulting in disinhibition and heightened nociception [51]. Spinal nerve ligation (SNL) models support this concept, showing approximately 16% loss of EGFP-labeled GABAergic neurons in the ipsilateral dorsal horn of GAD67-EGFP mice, which is reversed by antioxidant administration, implicating oxidative stress in GABAergic neuron vulnerability [52]. While most of these studies focused on males, emerging evidence suggests sex-specific modulation of GABAergic circuits. In spared nerve injury models, maladaptive GABAergic alterations in cortical regions differ by sex [53], and neuroimmune signaling via BDNF, resulting in downregulation of KCC2 and STEP61, occurs predominantly in males, with females showing resistance to such coupling [54]. These observations parallel our findings, underscoring that hyperexcitability pathways are differentially recruited across sexes.

Second, we show that the loss of GABAergic neurons in males is closely accompanied by increased ΔFosB expression, a coupling not previously documented in pain models. ΔFosB, a truncated and highly stable splice variant of FosB, functions as a persistent transcriptional regulator of long-term neural plasticity. In contrast to the rapid and transient expression of c-Fos [55], ΔFosB accumulates progressively with sustained stimulation [56], making it a marker of chronic, rather than acute, nociceptive activity. Although elevated ΔFosB has been extensively described in epilepsy models characterized by compromised GABAergic inhibition [57], its association with inhibitory neuronal loss in chronic pain states has not been previously recognized. Within mesolimbic regions such as the nucleus accumbens, ΔFosB accumulation contributes to mechanical allodynia and depressive-like behaviors [58, 59], while its upregulation in the medial prefrontal cortex is specifically linked to persistent pain and stress, but not acute injury [60]. In the central amygdala, ΔFosB serves as an integrative node for affective and nociceptive processes [61]. Together, these observations support our interpretation that ΔFosB functions as a transcriptional hub through which chronic pain reshapes inhibitory and emotional circuits, particularly in males.

Third, we identify a male-specific upregulation of stress-related neuropeptides, particularly PACAP and its downstream effectors, following chronic migraine induction. PACAP, widely expressed in stress-responsive regions such as the BNST, amygdala, hippocampus, and prefrontal cortex, signals through PAC1 to activate cAMP/PKA, MAPK/ERK, and CREB pathways, regulating neuroplasticity and survival [62–65]. PACAP can induce BDNF via CREB, influencing TrkB-dependent synaptic remodeling [65]. Critically, PACAP expression is sexually dimorphic: repeated stress increases PACAP mRNA in the BNST of males, but not females - in a testosterone-dependent manner [66–68]. Chronic stress models, including repeated variate stress and social defeat, robustly increase PACAP, PAC1, BDNF, and TrkB expression in males, correlating with heightened anxiety-like behavior [69, 70], while females show blunted responses, potentially due to estrogenic buffering or differential glucocorticoid signaling [71, 72]. These mechanisms may extend to chronic migraine, revealing hormone-sensitive stress circuitry in males.

Fourth, we identify dysregulation of GABAergic signaling within the choroid plexus (CP), a non-neuronal yet neuroactive interface increasingly implicated in migraine chronification. Traditionally regarded as a CSF-producing structure [73], the CP actively participates in immune modulation, metabolic regulation, and CSF ionic homeostasis [74, 75], with altered structure reported in migraine patients [76–78]. GABAergic machinery, including GAD65/67, GABA transporters, and receptor subunits, is expressed in CP epithelial cells [79]. The developmental “GABA switch” mediated by NKCC1 and KCC2, determines the polarity of GABA signaling [80–82]. Dysregulation of this transition is implicated in several neurological disorders [83], and may contribute to pathophysiological mechanisms relevant to migraine, particularly in chronic migraine, and migraine with aura. Dysregulated NKCC1/KCC2 expression within the CP may shift GABAergic signaling toward a depolarizing, excitatory state, promoting neuroinflammation and CSF-mediated sensitization [84]. These findings suggest that GABA signaling in the choroid plexus may function as a gatekeeper of CSF ionic composition and contribute to migraine-associated hypersensitivity via non-synaptic mechanisms. As such, the choroid plexus represents a previously underappreciated node in migraine chronification, particularly via GABA-chloride signaling pathways.

Our findings reveal a male-specific vulnerability of GABAergic neurons in chronic migraine, highlighting a fundamental sex-dependent divergence in underlying neurobiological mechanisms (Fig. 5). While migraine research has historically focused on excitatory signaling and neuropeptides such as calcitonin gene-related peptide (CGRP), our data point to disruption of inhibitory GABAergic circuits in males as a distinct and underexplored contributor to migraine chronification. Despite similar behavioral outcomes in males and females, this dissociation reflects sexually dimorphic mechanisms leading to convergent phenotypes [85, 86], with males exhibiting reduced inhibitory tone and females likely engaging alternative pathways, including enhanced parasympathetic regulation or distinct neuroimmune mechanisms, to achieve comparable behavioral endpoints. Notably, sex differences in autonomic cardiovascular regulation - males showing greater sympathetic-mediated responses and females enhanced parasympathetic tone, particularly under stress or pathological conditions, may further shape how chronic migraine manifests across sexes, influencing pain perception, neuroinflammation, and cerebrovascular dynamics [87, 88]. It is also possible that the comparable behavioral outcomes observed across sexes reflect a ‘ceiling effect’, particularly in females, whose nociceptive thresholds may reach a lower measurable limit. In such cases, additional neurobiological alterations may not produce further detectable behavioral change, thereby obscuring underlying mechanistic differences despite equivalent pain phenotypes.

### Limitations

Although female-specific changes were not the primary focus of this study, future investigations into cardiovascular and autonomic adaptations may provide critical insights, particularly given females’ greater susceptibility to migraine-associated cerebrovascular complications, including elevated stroke risk [89]. Additionally, while we restricted our analysis to four brain regions, it remains to be determined whether truly unaffected regions exist in the male brain, as chronic migraine is known to exert widespread effects across sensory, motor, visual, auditory, and other neural systems. Expanded regional mapping will be essential to define the full extent of inhibitory circuit disruption.

While our study employed the repeated nitroglycerin (NTG) model, several observations support that the GABAergic vulnerability we report is unlikely to result from nonspecific NTG toxicity. Both male and female mice developed comparable migraine-like hypersensitivity; however, only males exhibited GABAergic neuronal dysfunction, neuronal stress responses, and cell loss. Notably, there is currently no direct evidence of apoptosis in human male chronic migraine brains, and thus our findings should be interpreted as mechanistic rather than pathological confirmation. The absence of these alterations in females, despite identical NTG exposure, suggests a biologically selective and behaviorally relevant vulnerability rather than a general pharmacologic effect. Nevertheless, future studies employing complementary migraine paradigms, such as CGRP-based or genetic models will be necessary to determine whether this GABAergic susceptibility generalizes across migraine etiologies.

## Conclusion

Collectively, the identification of GABAergic dysfunction as a sexually divergent feature of chronic migraine expands our understanding of the disorder’s pathophysiological landscape and underscores the importance of dissecting molecular and circuit-level mechanisms in a sex-specific manner. Sex-specific susceptibility may involve hormonal or epigenetic factors, consistent with clinical observations that migraine pathophysiology differs between men and women. Male-specific GABAergic neuronal loss may enhance excitability in migraine-relevant brain regions, contributing to chronic migraine susceptibility, and highlights inhibitory interneurons and neuronal stress pathways as potential sex-specific therapeutic targets. Although both sexes develop behavioral hypersensitivity, the absence of GABAergic alterations in females suggests distinct mechanistic pathways underlying migraine-like behaviors. Developing sex-specific therapeutic strategies that target the distinct molecular vulnerabilities in male versus female patients may hold promise for improving treatment efficacy and personalized care in this highly heterogeneous disorder.

## Supplementary Information

Below is the link to the electronic supplementary material.

Supplementary Material 1

Supplementary Material 2

## Data Availability

No datasets were generated or analysed during the current study.
