# Supplementary material for: Selective vulnerability of GABAergic neurons in chronic migraine
Source: J Headache Pain. 2025 Nov 20;26(1):266. doi: 10.1186/s10194-025-02223-9 (PMC12636155; doi:10.1186/s10194-025-02223-9)
Supplement: Supplementary file 1 — Supplementary Material 1 [file 10194_2025_2223_MOESM1_ESM.pdf]

Selective Vulnerability of GABAergic Neurons in Chronic Migraine

Kazi Helal Hossain[1], Timothy Chuong[1], Emily Abad[1], Justin Lin[1], Chenchen Xia[2], Meng Li[3], Yibu Chen[3], Xianghong Arakaki[2ϕ], and Anju Vasudevan[1 ϕ]

[1]Angiogenesis and Brain Development Laboratory, Department of Neurosciences, Huntington Medical Research Institutes (HMRI), 686 S Fair Oaks Avenue, Pasadena, CA 91105, USA.

[2]Cognition and Brain Integration Laboratory, Department of Neurosciences, Huntington Medical Research Institutes (HMRI), 686 S Fair Oaks Avenue, Pasadena, CA 91105, USA.

[3]USC Libraries Bioinformatics, University of Southern California (USC), Health Science Campus, 2003 Zonal Ave, Los Angeles, CA 90089, USA.

[ϕ] Correspondence should be addressed to:
X.A. (xianghong.arakaki@hmri.org), or A.V. (anju.vasudevan@hmri.org)

Supplementary Figure 1

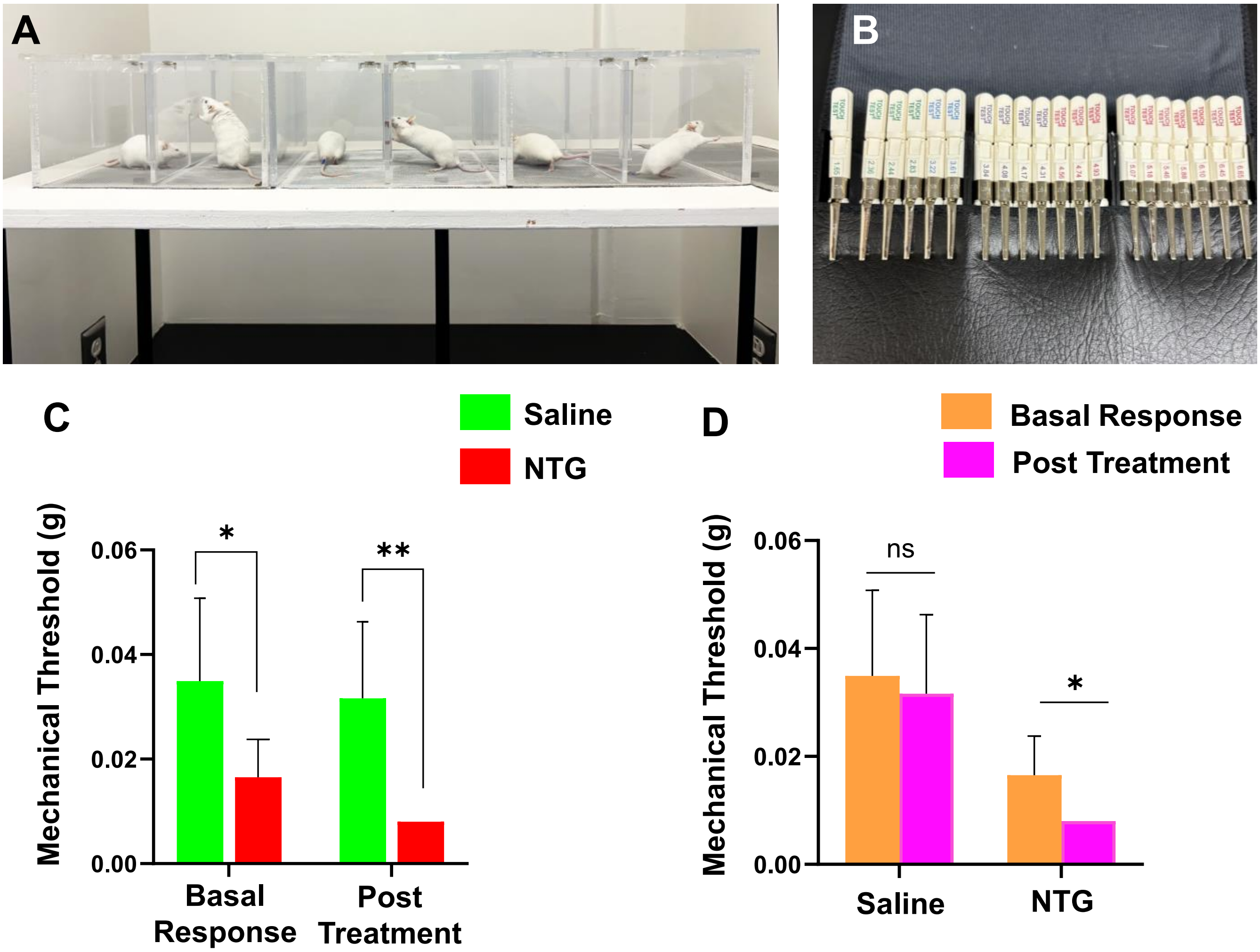

Supplementary Figure 1: NTG treatment induces mechanical hypersensitivity in GAD65-GFP mice, modeling chronic migraine-associated nociception.

Adult GAD65-GFP mice were administered intraperitoneal injections of either saline or nitroglycerin (NTG; 10 mg/kg) every other day for 9 days (total of five injections). (A) Representative image of GAD65-GFP mice undergoing von Frey testing. (B) Mechanical sensitivity was assessed using von Frey filaments. (C) NTG-treated mice (red) exhibited significantly increased mechanical sensitivity both at baseline and following the fifth NTG dose, compared to saline-treated controls (green). Data represents mean ± SD (n = 5 mice; *P=0.0459, basal response; **P=0.0070, post treatment, Student's t-test). (D) Notably, sensitivity was significantly greater after the fifth NTG dose (orange) compared to pre-treatment baseline (pink) within the NTG group. Data represents mean ± SD (n = 5 mice; *P=0.0295, Student's t-test). In contrast, saline-treated mice showed no significant difference between pre-treatment baseline (pink) and post-treatment (orange) responses unlike the NTG group. These findings confirm that repeated NTG administration induces robust mechanical hypersensitivity in GAD65-GFP mice, consistent with a chronic migraine phenotype.

Supplementary Figure 2

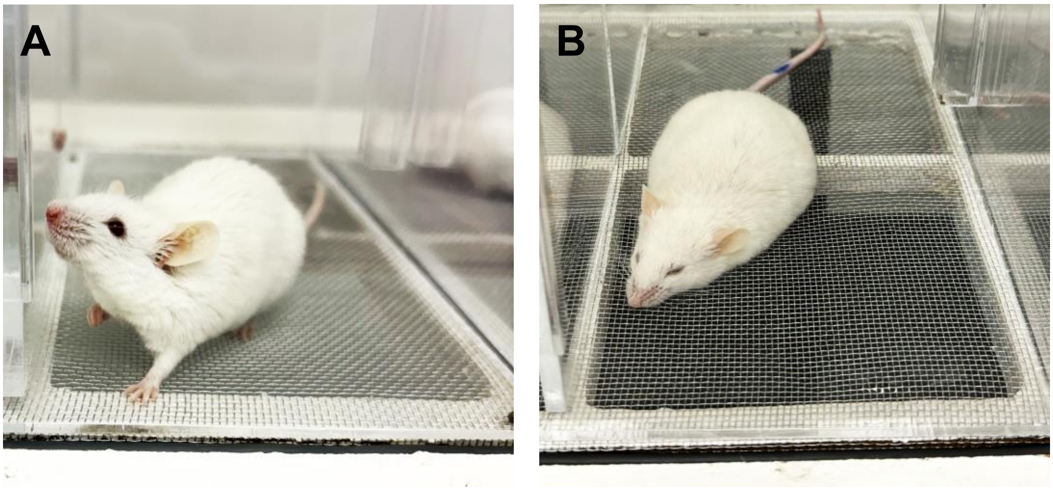

Saline NTG

Supplementary Figure 2: Elevated grimace response in GAD65-GFP mice following chronic NTG treatment.
Spontaneous grimacing is a behavioral indicator of nociception. (A) Representative image of a saline-treated GAD65-GFP mouse taken 60 minutes post-injection, showing a normal orbital appearance. (B) Representative image of an NTG-treated GAD65-GFP mouse 60 minutes after NTG administration (10 mg/kg, i.p.), displaying pronounced orbital tightening - a hallmark feature of the grimace response. These images are presented qualitatively to illustrate the characteristic nociceptive facial features associated with chronic NTG exposure.

Supplementary Figure 3

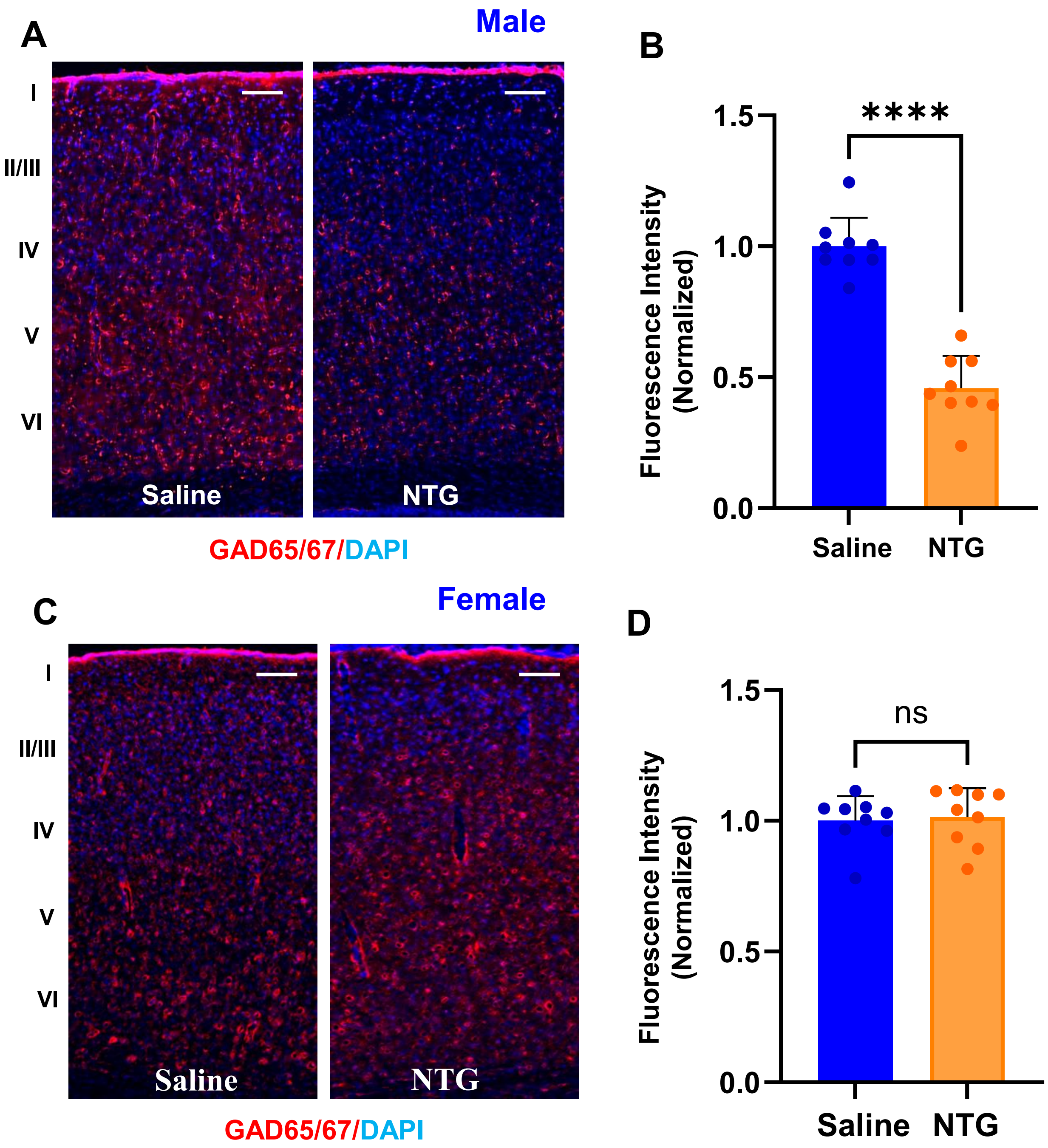

Supplementary Figure 3: GABAergic neuron loss is specific to male mice in the chronic migraine model.

GAD65 and GAD67 are the enzymes that synthesize GABA. To assess GABAergic neurons in the neocortex, anti-GAD65/67 immunohistochemistry with DAPI counterstaining was performed. (A, B) A significant reduction in GAD65/67$^+$ cells was observed in the somatosensory cortex of NTG-treated male mice compared to saline-treated controls, as quantified in (B). Data represent mean ± SD (n = 9 sections from 3 mice, ****$P<0.0001$, in Student's t-test). (C, D) In contrast, no reduction in GAD65/67$^+$ cells was detected in NTG-treated female mice compared to their respective controls, as shown in (C) and quantified in (D, n = 9 sections from 3 mice). These findings indicate a sex-specific vulnerability of GABAergic neurons to chronic migraine in male mice. Scale bars: 100 μm (A; applies to C).

Supplementary Figure 4

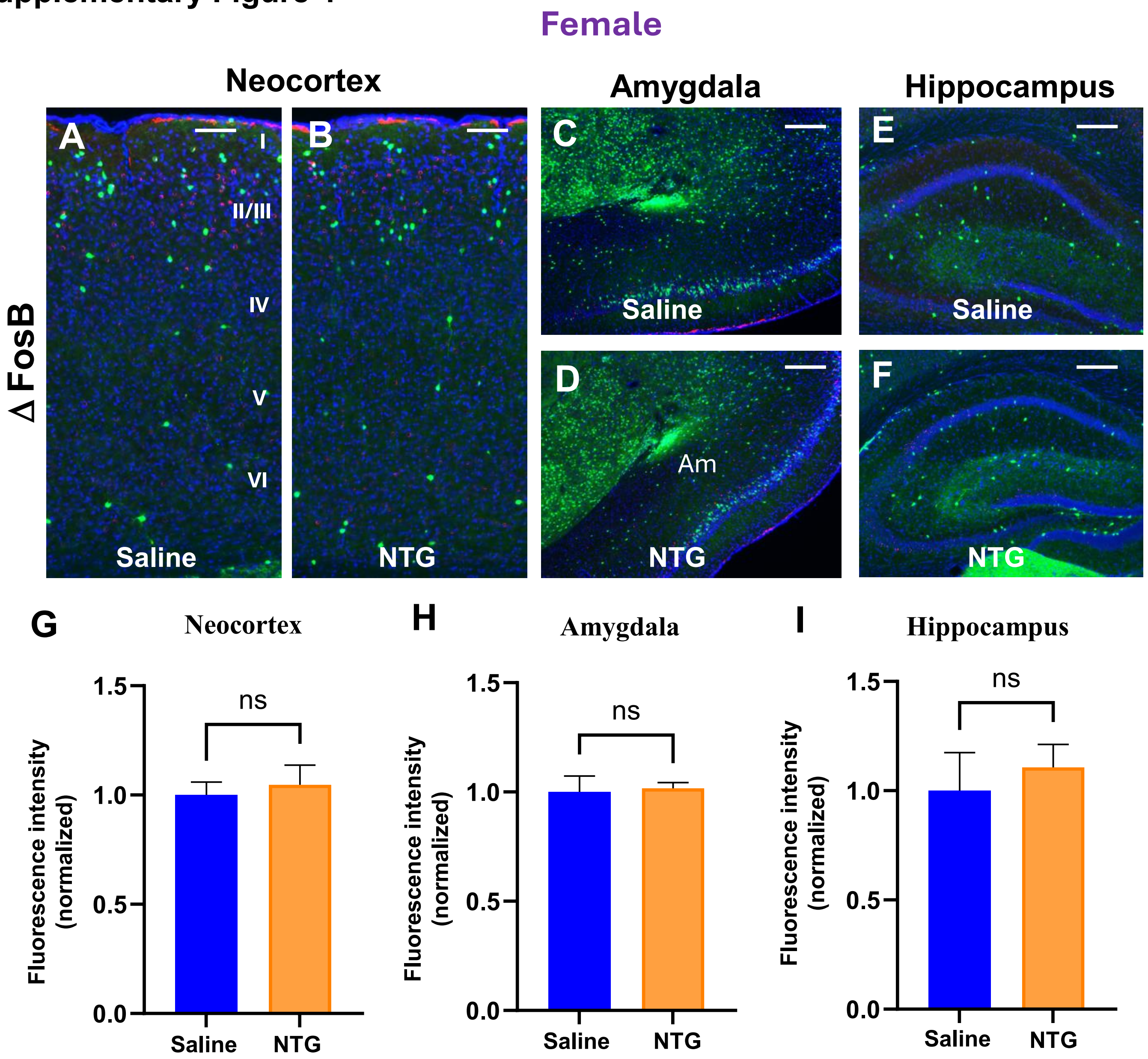

Supplementary Figure 4: Neuronal activity remains unchanged in female GAD65-GFP mice under chronic migraine conditions.

(A-F) Immunohistochemistry was performed to assess sustained neuronal activation in female GAD65-GFP mice using anti-ΔFosB (red) as a marker of prolonged neuronal activity and DAPI (blue) for nuclear staining. (G, H) Quantitative analysis revealed no significant differences in ΔFosB expression in the neocortex, amygdala, or hippocampus between NTG-treated and saline-treated female mice. Data represents mean ± SD (*n*=3 mice), suggesting that chronic NTG treatment does not elicit prolonged neuronal activation in these regions in female mice. Scale bars: 100 μm (A; applies to B-F).

Supplementary Figure 5

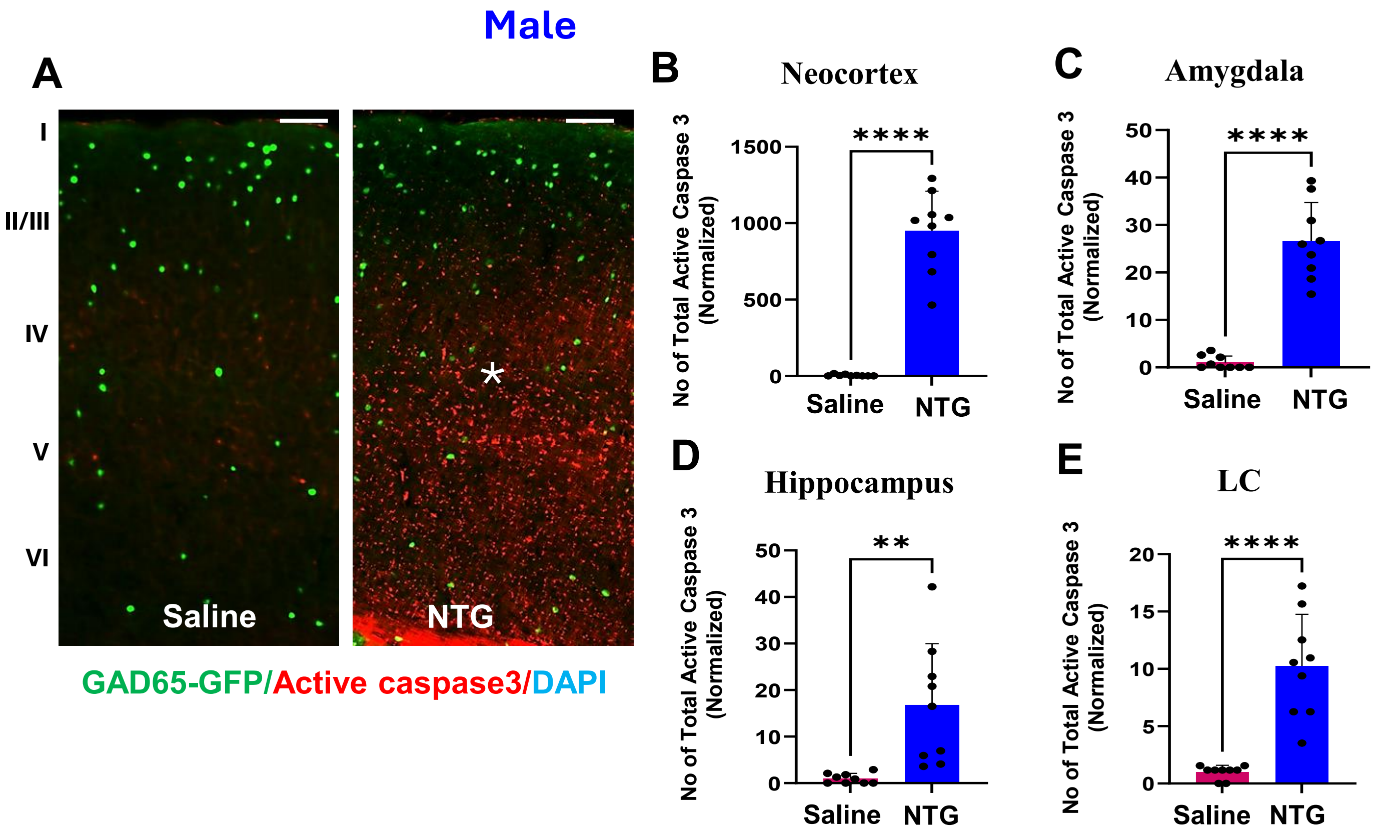

Supplementary Figure 5: Elevated apoptotic signaling in male mice with chronic NTG-induced migraine.

(A) Apoptosis was assessed by immunohistochemical detection of active caspase-3. Representative image of the somatosensory cortex (asterisks) shows increased active caspase-3 expression in NTG-treated mice compared to saline-treated controls. (B-E) Quantification of active caspase-3 expression revealed a significant increase in the neocortex (B), amygdala (C), hippocampus (D), and locus coeruleus (E) in NTG-treated males relative to controls. Data represent mean ± SD (*n* = 9 sections from 3 mice; ****P<0.0001, **P=0.0024, Student's *t*-test), indicating enhanced apoptotic signaling across multiple brain regions in the male chronic migraine model. Scale bars: 100 µm (A).

Supplementary Figure 6

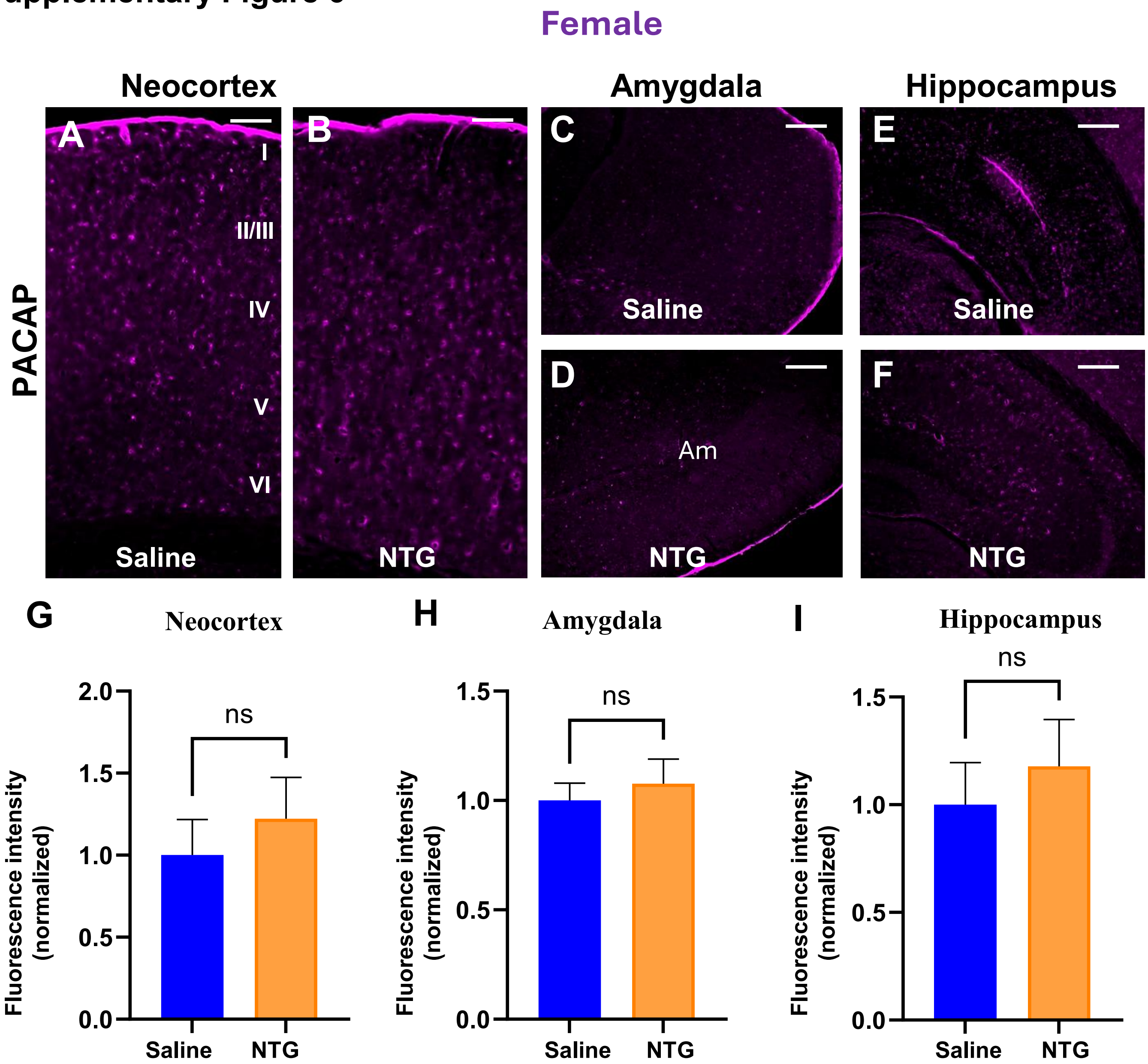

Supplementary Figure 6: Stress-related PACAP signaling remains unaltered in female GAD65-GFP mice with chronic NTG-induced migraine.
(A-F) Immunohistochemical analysis of PACAP (pituitary adenylate cyclase-activating polypeptide; purple), a neuropeptide associated with stress responses, was performed to evaluate stress signaling in female GAD65-GFP mice under chronic migraine conditions. (G-I) Quantification of PACAP expression in the neocortex, amygdala, and hippocampus revealed no significant differences between NTG-treated and saline-treated female mice.
Data represents mean ± SD (n = 3 mice), indicating that chronic NTG administration does not alter PACAP-mediated stress signaling in female mice. Scale bars: 100 µm (A; applies to B-F).

Supplementary Figure 7

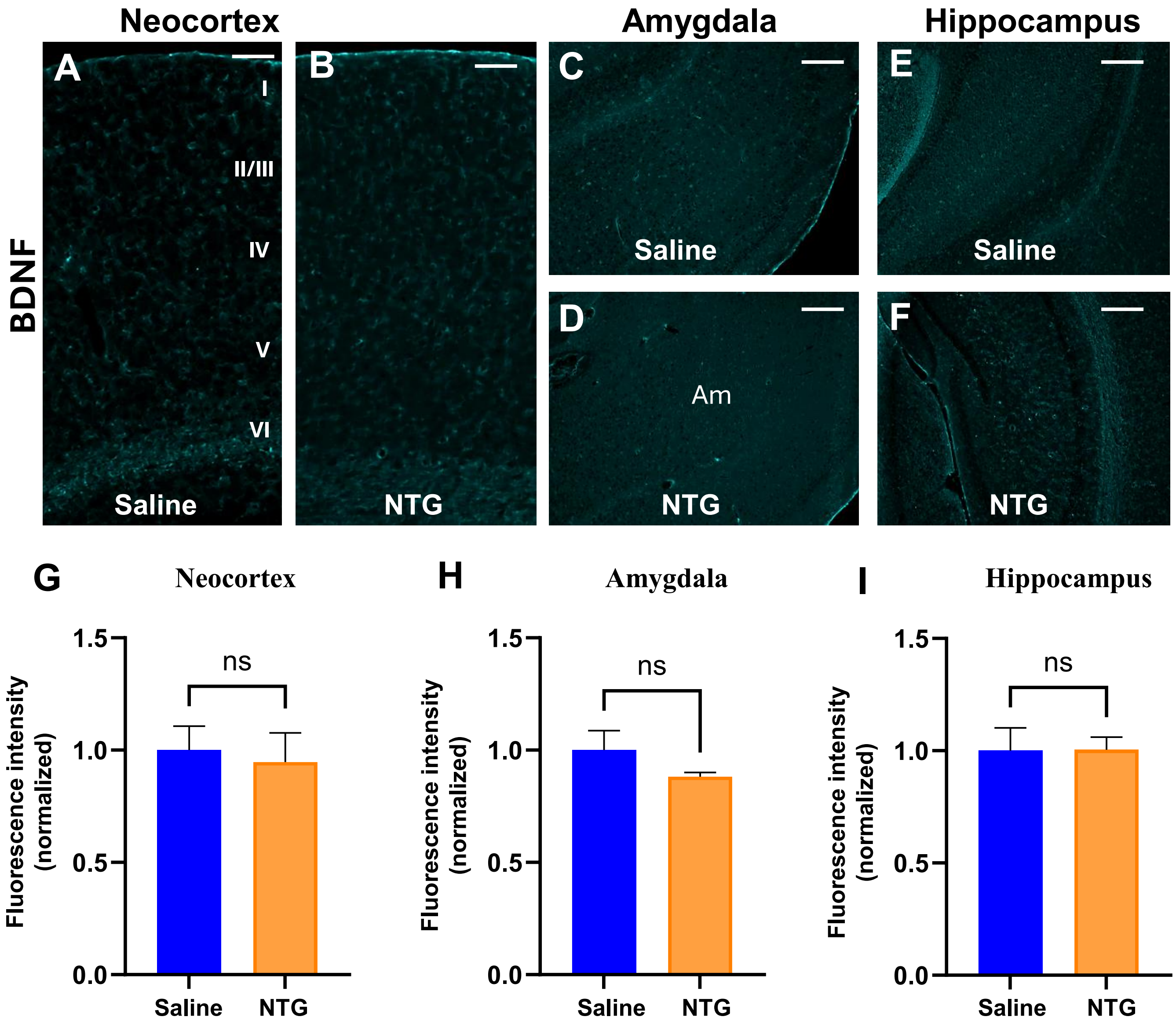

Supplementary Figure 7: BDNF, a downstream effector of stress signaling, is unchanged in female GAD65-GFP mice with chronic migraine.

(A-F) Immunohistochemistry using anti-BDNF (aqua) was performed to assess expression of brain-derived neurotrophic factor (BDNF), a key downstream mediator of stress signaling, in female GAD65-GFP mice under chronic NTG-induced migraine conditions. (H–I) Quantitative analysis revealed no significant differences in BDNF levels in the neocortex, amygdala, or hippocampus between NTG-treated and saline-treated female mice. Data represents mean ± SD (n = 3 mice), indicating that BDNF-mediated stress signaling is not altered in female mice following chronic NTG exposure. Scale bars: 100 µm (A; applies to B-F).

Supplementary Figure 8

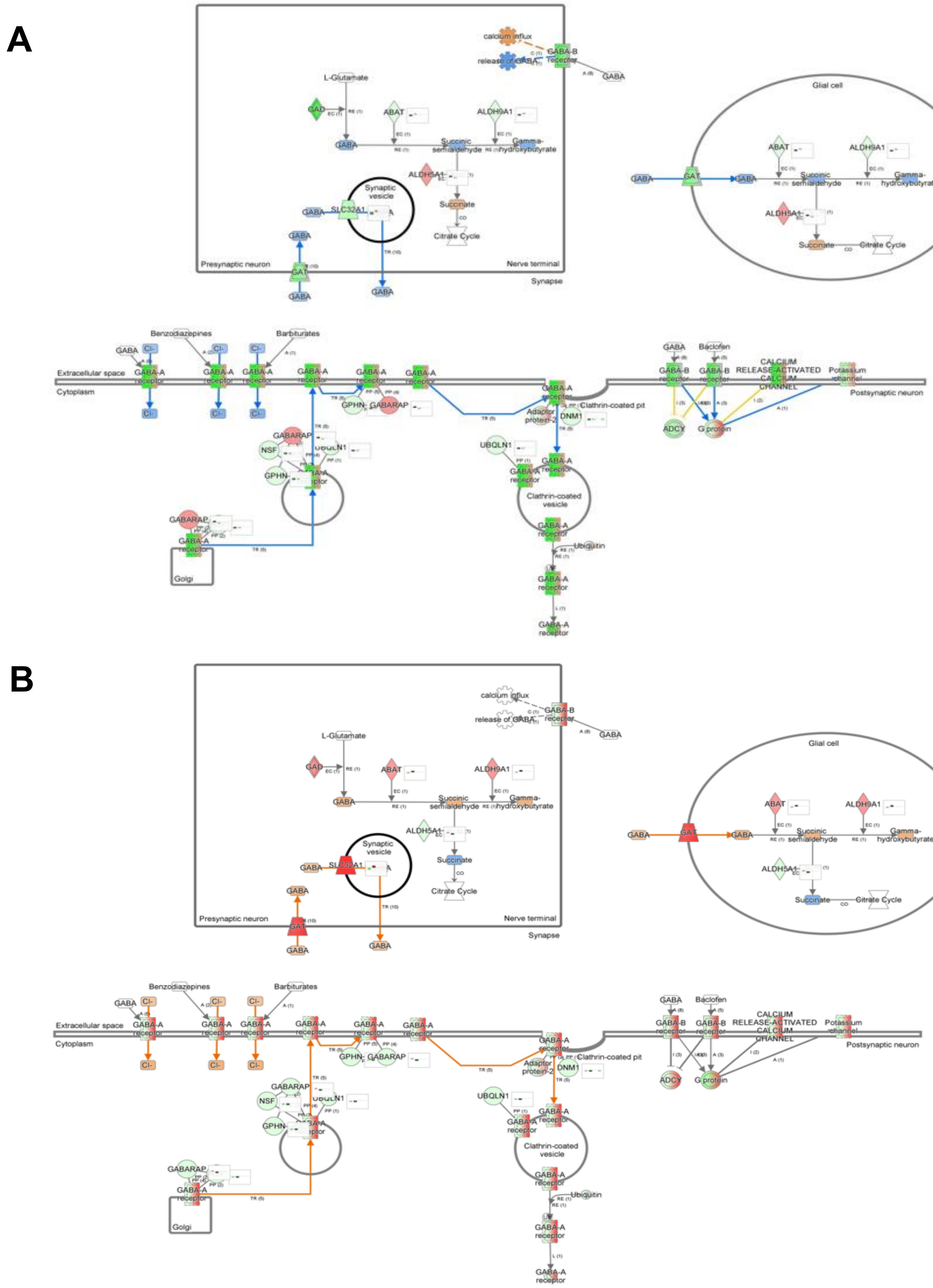

Supplementary Figure 8: Sex-specific regulation of GABA receptor signaling pathways in response to chronic NTG treatment.

(A, B) Ingenuity Pathway Analysis (IPA) of gene expression data revealed differential regulation of GABA receptor signaling in male and female mice following NTG treatment. Pathway overlay illustrates a downregulation of GABA signaling genes in males (green, A) and a corresponding upregulation in females (orange, B), highlighting a sex-dependent divergence in GABAergic pathway activation.

Supplementary Figure 9

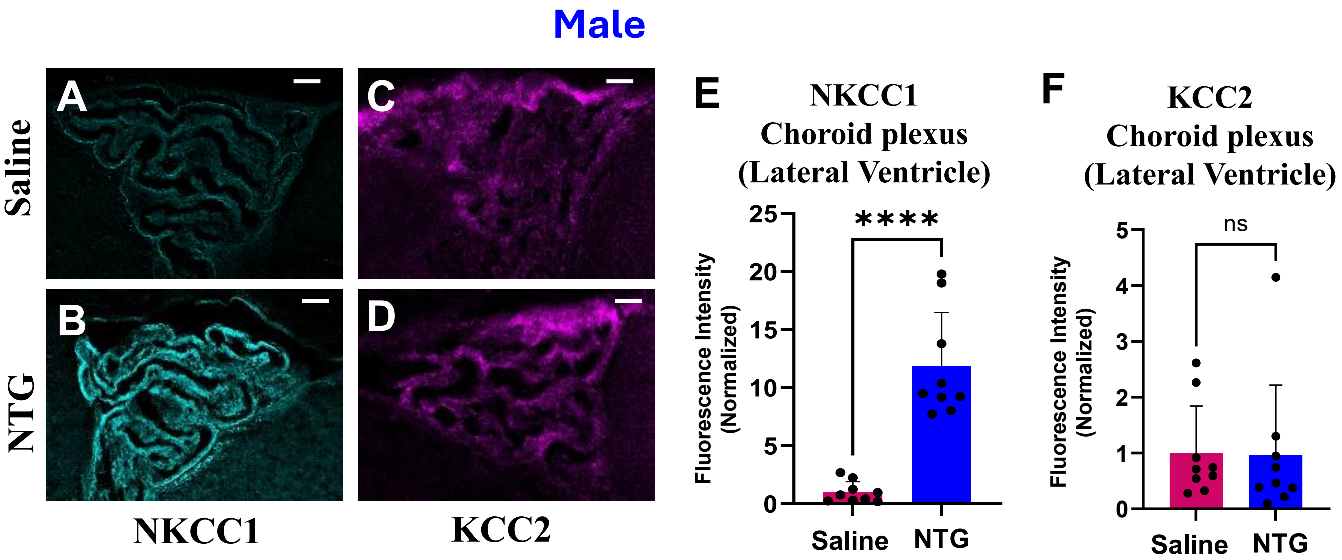

Supplementary Figure 9: Aberrant overexpression of the chloride cotransporter NKCC1 in the lateral ventricle choroid plexus in male GAD65-GFP mice with chronic migraine.

NKCC1 and KCC2 are chloride cotransporters that regulate intracellular chloride homeostasis and influence neuronal excitability - NKCC1 facilitates Cl^- influx, while KCC2 mediates Cl^- efflux. (A-D) To evaluate GABAergic signaling dynamics in male GAD65-GFP mice under chronic NTG-induced migraine conditions, immunohistochemistry was performed using anti-NKCC1 (aqua) and anti-KCC2 (purple) antibodies. (B) NTG-treated mice exhibited elevated NKCC1 expression in the choroid plexus of the lateral ventricle compared to saline-treated controls (A), with quantification shown in (E, Data represent mean ± SD, n = 9 sections from 3 mice; ****$P<0.0001$, Student's t-test). (C, D) In contrast, KCC2 expression remained unchanged between NTG- and saline-treated groups, as quantified in F. These findings suggest disrupted chloride homeostasis via NKCC1 upregulation in male chronic migraine mice. Scale bars: 50 µm (A; applies to B-D).
