# Supplementary material for: Selective vulnerability of GABAergic neurons in chronic migraine
Source: J Headache Pain. 2025 Nov 20;26(1):266. doi: 10.1186/s10194-025-02223-9 (PMC12636155; doi:10.1186/s10194-025-02223-9)
Supplement: Supplementary file 2 — Supplementary Material 2 [file 10194_2025_2223_MOESM2_ESM.pdf]

Supplementary Table 1: Sample-size calculation and power analysis

A. GAD65-GFP fluorescence (Figure 1)

The primary outcome was GAD65-GFP fluorescence in a defined brain region, averaged across three anatomically matched sections in the left and right hemispheres per mouse. Preliminary data indicated robust sex-specific responses; therefore, analyses and power calculations were performed separately for males and females.

Males: Pilot data revealed striking group differences (Saline: 1.00 ± 0.06 vs. NTG: 0.23 ± 0.02), corresponding to an exceptionally large effect size (Cohen's $d \approx 18$). Based on this effect, n = 3 mice per group was sufficient to achieve near 100% statistical power at $\alpha = 0.05$. This sample size was selected to balance statistical rigor with ethical reduction of animal use.

Females: Pilot analyses in females did not reveal a measurable effect between saline and NTG treatments. Nevertheless, n = 3 per group was retained to maintain consistency, enable exploratory comparisons, and support transparency and reproducibility in reporting.

B. Immunohistochemistry experiments (Figures 2-5)

Power calculations were performed separately by sex because preliminary data indicated sex-specific responses. For males, pilot data (n = 3 per group) yielded means of 1.00 ± 0.31 (Saline) and 8.67 ± 1.67 (NTG), pooled SD = 1.20 and Cohen's d ≈ 6.38. Based on this effect, n = 3 mice per group was sufficient to achieve near 100% statistical power at $\alpha = 0.05$. Therefore, we retained n = 3 per group for males to provide robust biological replication while minimizing animal use. For females, no clear pilot effect was detected; and n = 3 per group was maintained for transparency sex-balanced reporting and reproducibility.

Supplementary Table 2 - Multiple Comparisons After Two-Way ANOVA of Fig. 1E

Brain Region	Comparison	Mean Diff.	95% CI of Diff.	P Value Tukey	P Value BH	Significance Tukey/BH
Neocortex	NTG vs. Saline	-0.7716	-1.003 to -0.5402	<0.0001	<0.0001	Yes/Yes
Amygdala	NTG vs. Saline	-0.8631	-1.095 to -0.6317	<0.0001	<0.0001	Yes/Yes
Hippocampus	NTG vs. Saline	-0.9481	-1.180 to -0.7167	<0.0001	<0.0001	Yes/Yes
Locus Coeruleus	NTG vs. Saline	-0.9249	-1.156 to -0.6935	<0.0001	<0.0001	Yes/Yes

Supplementary Table 2: GAD65-GFP cellular data across different brain regions were analyzed using linear mixed-effects models. A two-way ANOVA with factors Treatment and Region was performed for each outcome. Pairwise post-hoc contrasts were adjusted using Tukey's test. In addition, we applied for Benjamini-Hochberg (BH) FDR correction across all readout × region pairwise tests. Significant reductions in the NTG versus saline groups were observed across all four regions (adjusted $p<0.0001$ for all comparisons).

Supplementary Table 3 - Multiple Comparisons After Two-Way ANOVA of Fig. 2I-L

Brain Region	Comparison	Mean Diff.	95% CI of Diff.	P Value Tukey	P Value BH	Significance Tukey/BH
Neocortex	NTG vs. Saline	7.674	5.548 to 9.799	<0.0001	<0.0001	Yes/Yes
Amygdala	NTG vs. Saline	1.772	-0.3531 to 3.898	0.1687	0.0111	No/Yes
Hippocampus	NTG vs. Saline	4.176	2.050 to 6.301	<0.0001	<0.0001	Yes/Yes
Locus Coeruleus	NTG vs. Saline	4.497	2.371 to 6.622	<0.0001	<0.0001	Yes/Yes

Supplementary Table 3: ΔFosB immunofluorescence data across different brain regions were analyzed using linear mixed-effects models. A two-way ANOVA with factors Treatment and Region was performed for each outcome. Pairwise post-hoc contrasts were adjusted using Tukey's test. In addition, we applied for Benjamini-Hochberg (BH) FDR correction across all readout × region pairwise tests. Significant increases in the NTG versus saline groups were observed in the neocortex, hippocampus, and locus coeruleus (adjusted $p<0.0001$ for all comparisons). In the amygdala, significance was observed only with the Benjamini-Hochberg FDR correction.

Supplementary Table 4 - Multiple Comparisons After Two-Way ANOVA of Fig. 2Q-T

Brain Region	Comparison	Mean Diff.	95% CI of Diff.	P Value Tukey	P Value BH	Significance Tukey/BH
Neocortex	NTG vs. Saline	83.19	40.26 to 126.1	<0.0001	<0.0001	Yes/Yes
Amygdala	NTG vs. Saline	52.69	9.762 to 95.61	0.0066	0.0003	Yes/Yes
Hippocampus	NTG vs. Saline	15.83	-27.10 to 58.75	0.9376	0.2507	No/No
Locus Coeruleus	NTG vs. Saline	20.33	-22.59 to 63.26	0.8087	0.1415	No/No

Supplementary Table 4: Active Caspase 3 data across different brain regions were also re-analyzed using linear mixed-effects models. A two-way ANOVA with factors Treatment and Region was performed for each outcome. Pairwise post-hoc contrasts were adjusted using Tukey's test. In addition, we applied for Benjamini-Hochberg (BH) FDR correction across all readout × region pairwise tests. Significant increases in the NTG versus saline groups were observed in the neocortex, and amygdala. No significance was observed in the hippocampus and locus coeruleus.

Supplementary Table 5 - Multiple Comparisons After Two-Way ANOVA of Fig. 3I-L

Brain Region	Comparison	Mean Diff.	95% CI of Diff.	P Value Tukey	P Value BH	Significance Tukey/BH
Neocortex	NTG vs. Saline	1.31	0.1992 to 2.063	0.0076	0.0003	Yes/Yes
Amygdala	NTG vs. Saline	2.773	1.841 to 3.705	<0.0001	<0.0001	Yes/Yes
Hippocampus	NTG vs. Saline	2.394	1.462 to 3.326	<0.0001	<0.0001	Yes/Yes
Locus Coeruleus	NTG vs. Saline	2.236	1.304 to 3.168	<0.0001	<0.0001	Yes/Yes

Supplementary Table 5: PACAP fluorescence data across different brain regions were also re-analyzed using linear mixed-effects models. A two-way ANOVA with factors Treatment and Region was performed for each outcome. Pairwise post-hoc contrasts were adjusted using Tukey's test. In addition, we applied for Benjamini-Hochberg (BH) FDR correction across all readout × region pairwise tests. Significant increases in the NTG versus saline groups were observed across all four regions.

Supplementary Table 6 - Multiple Comparisons After Two-Way ANOVA of Fig. 3M-P

Brain Region	Comparison	Mean Diff.	95% CI of Diff.	P Value Tukey	P Value BH	Significance Tukey/BH
Neocortex	NTG vs. Saline	3.278	2.096 to 4.460	<0.0001	<0.0001	Yes/Yes
Amygdala	NTG vs. Saline	3.277	2.094 to 4.459	<0.0001	<0.0001	Yes/Yes
Hippocampus	NTG vs. Saline	1.508	0.3256 to 2.690	0.0042	0.0002	Yes/Yes
Locus Coeruleus	NTG vs. Saline	2.022	0.8395 to 3.024	<0.0001	<0.0001	Yes/Yes

Supplementary Table 6: PAC1 fluorescence data across different brain regions were also re-analyzed using linear mixed-effects models. A two-way ANOVA with factors Treatment and Region was performed for each outcome. Pairwise post-hoc contrasts were adjusted using Tukey's test. In addition, we applied for Benjamini-Hochberg (BH) FDR correction across all readout × region pairwise tests. Significant increases in the NTG versus saline groups were observed across all four regions.

Supplementary Table 7 - Multiple Comparisons After Two-Way ANOVA of Fig. 4I-L

Brain Region	Comparison	Mean Diff.	95% CI of Diff.	P Value Tukey	P Value BH	Significance Tukey/BH
Neocortex	NTG vs. Saline	4.523	2.650 to 6.397	<0.0001	<0.0001	Yes/Yes
Amygdala	NTG vs. Saline	3.462	1.588 to 5.336	<0.0001	<0.0001	Yes/Yes
Hippocampus	NTG vs. Saline	7.883	6.010 to 9.757	<0.0001	<0.0001	Yes/Yes
Locus Coeruleus	NTG vs. Saline	2.794	0.9208 to 4.688	0.0004	<0.0001	Yes/Yes

Supplementary Table 7: BDNF fluorescence data across different brain regions were also re-analyzed using linear mixed-effects models. A two-way ANOVA with factors Treatment and Region was performed for each outcome. Pairwise post-hoc contrasts were adjusted using Tukey's test. In addition, we applied for Benjamini-Hochberg (BH) FDR correction across all readout × region pairwise tests. Significant increases in the NTG versus saline groups were observed across all four regions.

Supplementary Table 8 - Multiple Comparisons After Two-Way ANOVA of Fig. 4M-P

Brain Region	Comparison	Mean Diff.	95% CI of Diff.	P Value Tukey	P Value BH	Significance Tukey/BH
Neocortex	NTG vs. Saline	2.581	1.497 to 3.664	<0.0001	<0.0001	Yes/Yes
Amygdala	NTG vs. Saline	1.474	0.3906 to 2.558	0.0018	<0.0001	Yes/Yes
Hippocampus	NTG vs. Saline	1.257	0.1739 to 2.341	0.0125	0.0006	Yes/Yes
Locus Coeruleus	NTG vs. Saline	0.7832	-0.3003 to 1.867	0.3250	0.0267	No/Yes

Supplementary Table 8: TRK1B immunofluorescence data across different brain regions were also re-analyzed using linear mixed-effects models. A two-way ANOVA with factors Treatment and Region was performed for each outcome. Pairwise post-hoc contrasts were adjusted using Tukey's test. In addition, we applied for Benjamini-Hochberg FDR correction across all readout × region pairwise tests. Significant increases in the NTG versus saline groups were observed in the neocortex, amygdala, and hippocampus. In the locus coeruleus, significance was observed only with the Benjamini-Hochberg FDR correction.
